# Kinetics insight into the roles of the N- and C-lobes of calmodulin in RyR1 channel regulation

**DOI:** 10.1016/j.jbc.2025.108258

**Published:** 2025-02-02

**Authors:** Jingyan Zhang, Levy M. Treinen, Skylar J. Mast, Megan R. McCarthy, Bengt Svensson, David D. Thomas, Razvan L. Cornea

**Affiliations:** Department of Biochemistry, Molecular Biology, and Biophysics, University of Minnesota, Minneapolis, USA

**Keywords:** Ca^2+^ binding, calcium release channel, fluorescence labeling, oximimetic, ryanodine receptor

## Abstract

Calmodulin (CaM) activates the skeletal muscle Ca^2+^ release channel (ryanodine receptor, RyR1) at nanomolar Ca^2+^ and inhibits it at micromolar Ca^2+^. CaM conversion from RyR1 activator to inhibitor is due to structural changes induced by Ca^2+^ binding at CaM’s two lobes. However, it remains unclear which lobe provides the switch for this conversion. Here, we attached the environment-sensitive fluorophore acrylodan (Acr) at either lobe of intact CaM or lobe-specific Ca^2+^-sensitive CaM mutants, and monitored the effects of Ca^2+^ binding *via* the fluorescence change of free or RyR1-bound ^Acr^CaM. Using steady state measurements, we found that Ca^2+^ binding to free CaM causes a dramatic structural change in the N-lobe, but only a slight effect on the C-lobe of the Ca^2+^-sensitive lobe-specific mutants, in addition to the previously known higher Ca^2+^ affinity at the C-lobe *versus* the N-lobe. Using stopped-flow measurements, we found ∼30x faster Ca^2+^ dissociation from the N- *versus* C-lobe, and ∼20x slower Ca^2+^ association to the N-lobe *versus* C-lobe. These Ca^2+^ binding properties hold for the CaM/RyR1 complex, and Ca^2+^ affinity is enhanced at the CaM C-lobe but decreased at the N-lobe by RyR1 binding. We propose that fast Ca^2+^-binding at the C-lobe of CaM initiates its inhibition to RyR1 at high [Ca^2+^], while slow Ca^2+^ binding to the N-lobe is necessary for timely enhancement of the inhibitory effect. The dysregulation of RyR1 by M124Q-CaM may be explained by the lower Ca^2+^ affinity *versus* WT-CaM, as suggested by both steady-state and transient kinetics results.

Calmodulin (CaM) is an abundant and ubiquitous Ca^2+^-sensing protein that serves as one of the key regulators of the ryanodine receptor (RyR1), a major player of Ca^2+^ regulation in excitation-contraction (EC) coupling in skeletal muscle (1). CaM regulation of RyR1 is attributed to its unique structure, composed of two four-helix bundle domains (termed N- and C-lobes), each of which containing a pair of EF-hand sites that bind Ca^2+^ cooperatively (2). The two lobes of CaM exhibit different Ca^2+^ binding properties: the C-lobe has ∼10-fold higher affinity for Ca^2+^ than the N-lobe, resulting in sequential Ca^2+^ occupancy of the two lobes in solution (3, 4). Ca^2+^ binding at the two lobes of CaM also induces its structural rearrangements (5). CaM regulates RyR1 function in a [Ca^2+^]-dependent manner; it is an agonist of RyR1 activity at nanomolar [Ca^2+^] and an inhibitor at micromolar [Ca^2+^] (6, 7, 8). Ca^2+^-binding to CaM has been extensively studied, exploiting its native phenylalanine and tyrosine fluorescence. However, a CaM-binding peptide (CaMBP) has been persistently used as an RyR surrogate to evaluate Ca^2+^ binding to the CaM-RyR complex (4, 9, 10), despite evidence that CaMBP provides weak resemblance of the RyR CaM binding sites (11) Earlier studies proposed that CaM conversion from RyR1 activator to inhibitor is due to Ca^2+^ binding at two EF-hand motifs in each of the two lobes of CaM (5). Subsequent studies suggested that Ca^2+^ binding to the C-lobe converts CaM from an activator to an inhibitor, while interaction with the N-lobe is required for a maximum effect on RyR1 (12). However, further studies with CaMBP concluded that the occupancy of the N-lobe Ca^2+^ binding sites in CaM is responsible for the CaM-dependent modulation of the RyR1 calcium release channel (13). Studies with CaMBP provide valuable structural and mechanistic information of CaM regulation to RyR1. However the crystal structure of CaM/CaMBP depicts a binding mode that is quite different from the recent high-resolution cryo-EM structures of apo- and Ca^2+^-CaM/RyR2 (cardiac isoform) (14), apo-CaM/RyR1 (15, 16), and Ca^2+^-CaM/RyR1 (17). In RyR2 cryo-EM structures, apo-CaM assumes an extended conformation in both open and closed states of the channel (14). Ca^2+^-CaM’s binds to RyR2 in a location that is shifted downward toward the central domain and adopts a more compact conformation (14). This CaM conformation is similar to what has been observed in the crystal structure of CaM bound to CaMBP (18). In RyR1 cryo-EM structures, apo-CaM (15) and Ca^2+^-CaM (17) show similar binding locations. However, Ca^2+^-CaM shows a significant structural change within the N-lobe and a slight shift (translation) in the position of C-lobe toward the RyR1 helical domain. The existence of the two partially overlapping but distinct binding sites for CaM in RyR1 implies that Ca^2+^ binding to CaM switches its binding location (16) and regulatory role. Two coexisting conformations in CaM/RyR1 were also proposed based on fluorescence lifetime (FLT)-detected FRET measurements using covalently labeled CaM on each lobe with fluorescent probes, both in the presence and absence of RyR1 (19). FLT FRET in combination with trilateration (20) indicate a significant Ca^2+^-driven shift of RyR1-bound CaM that is more consistent with the Ca^2+^-CaM binding location observed in cryo-EM studies of RyR2. To date, the CaM/CaMBP structure has not been observed in cryo-EM studies of the CaM/RyR1 complex, thus challenging the field to develop new methods for observing the structural kinetics of CaM while bound to intact, functional RyRs, in their native membrane environment.

Cryo-EM of CaM/RyR provides structural snapshots that are essential for resolving the crucial roles of CaM as a regulatory partner of RyR, but how CaM kinetically regulates the open/closing of the channel of RyR through Ca^2+^ binding remains unknown. Early ^43^Ca^2+^-NMR studies reported a fast (∼1000 s^−1^) and a slow (20–50 s^−1^) Ca^2+^ off rate, which were attributed to the low- and high-affinity Ca^2+^ binding sites in the N- and C-lobes, respectively (21). ^l^H-NMR measurements obtained similarly fast (>600 s^−1^) and slow (<50 s^-l^) Ca^2+^ off rates (22). Early stopped-flow studies showed only a single Ca^2+^ dissociation rate of ∼12 s^-l^ using intrinsic tyrosine fluorescence (23, 24). More recently, stopped-flow studies using indicator Quin two as a Ca^2+^ chelator and a fluorescent probe at 11–28 °C indicated two rate constants, possibly corresponding to the Ca^2+^ dissociation from the two lobes (24, 25). Much faster off rates were also reported by directly determining the fall in [Ca^2+^]_free_ after a rapid change of total [Ca^2+^] produced by flash photolysis of DM-nitrophen (26). Surprisingly, using the same method, that study also showed that Ca^2+^ binds to the N-lobe of CaM faster than to the C-lobe (7.7 × 10^8^ M^−1^ s^−1^ and 3.2 × 10^10^ M^−1^ s^−1^) (26). By tagging CaM in the linker region and using microfluidic mixing and traditional stopped-flow kinetics, Park *et al.* measured conformational changes induced by Ca^2+^ binding and reported two rate constants (∼2000 s^−1^ and 50 s^−1^) that were attributed to Ca^2+^ binding to the C- and N-lobe, respectively (27). Accumulating evidence suggests that the kinetics of Ca^2+^ binding of CaM is also modulated by its interaction with target proteins, leading to CaM’s regulatory function being specifically tuned to each target (28). Therefore, measuring the kinetics of Ca^2+^ binding to RyR1-bound CaM is crucial for understanding CaM regulation of the RyR1 channel.

CaM regulation of RyR1 not only involves Ca^2+^ binding to CaM, but also CaM binding to RyR1. Given that both apo- and Ca^2+^-CaM can bind to RyRs with high affinity (nanomolar K_D_ values), and nanomolar free CaM exists in cells, most RyR should be in a CaM-bound state in cells, regardless of Ca^2+^ concentration (28, 29). Previously, we have shown increased Ca-binding affinity of CaM when in complex with RyR1 in heavy skeletal sarcoplasmic reticulum (HSR) (11). Recently, we used stopped flow in combination with FRET to monitor the association rates of CaM binding to RyR, as well as the faster rates of Ca^2+^-driven structural transition of CaM bound to RyR1 or RyR2 (20). FLT time course was acquired after rapid (2 ms) mixing with Ca^2+^ to increase [Ca^2+^] from 30 nM to μM, or EGTA to decrease [Ca^2+^] from 30 μM to nM. The results suggest that CaM shifts rapidly during EC coupling to become an RyR1 inhibitor, faster than its transition to become an RyR1 activator during relaxation.

Our goal in the current study is to understand lobe-specific CaM regulation of RyR1 by monitoring the kinetics of Ca^2+^-driven conformational changes of RyR1-bound CaM. We substituted cysteines within the C- or N-lobe portions of CaM (T34 and T110, respectively) to attach the environmentally sensitive fluorescent probe, acrylodan (Acr) (11, 13, 30). We also ablated the Ca^2+^ binding sites of the N-lobe (E31A/E67A) or C-lobe (E104A/E140A) to retain Ca^2+^ binding in the C-lobe (CaM_C_) or the N-lobe (CaM_N_), respectively (illustrated in Fig. 1 cartoon insets). In these lobe-specific Ca^2+^-sensitive CaM mutants, the environmentally sensitive fluorophore Acr is expected to selectively report the structural changes of one lobe of CaM to better understand the role of each lobe, as induced by Ca^2+^ binding to free and RyR1-bound CaM. Moreover, previous studies have shown that CaM methionine oxidation is disruptive to its secondary and tertiary structure and this correlates with strong effects on CaM’s ability to regulate RyRs (31). CaM contains nine methionines, all susceptible to oxidation (32). Of particular interest is M124 (18), whose oxidation abolishes regulation of both RyR1 and RyR2 (18, 31, 33). Our observations of the kinetics of Ca^2+^ binding suggest that dysregulation of RyR1 by the oximimetic CaM mutant M124Q probably originates from its intrinsically lower affinity for Ca^2+^.

**Figure 1:**
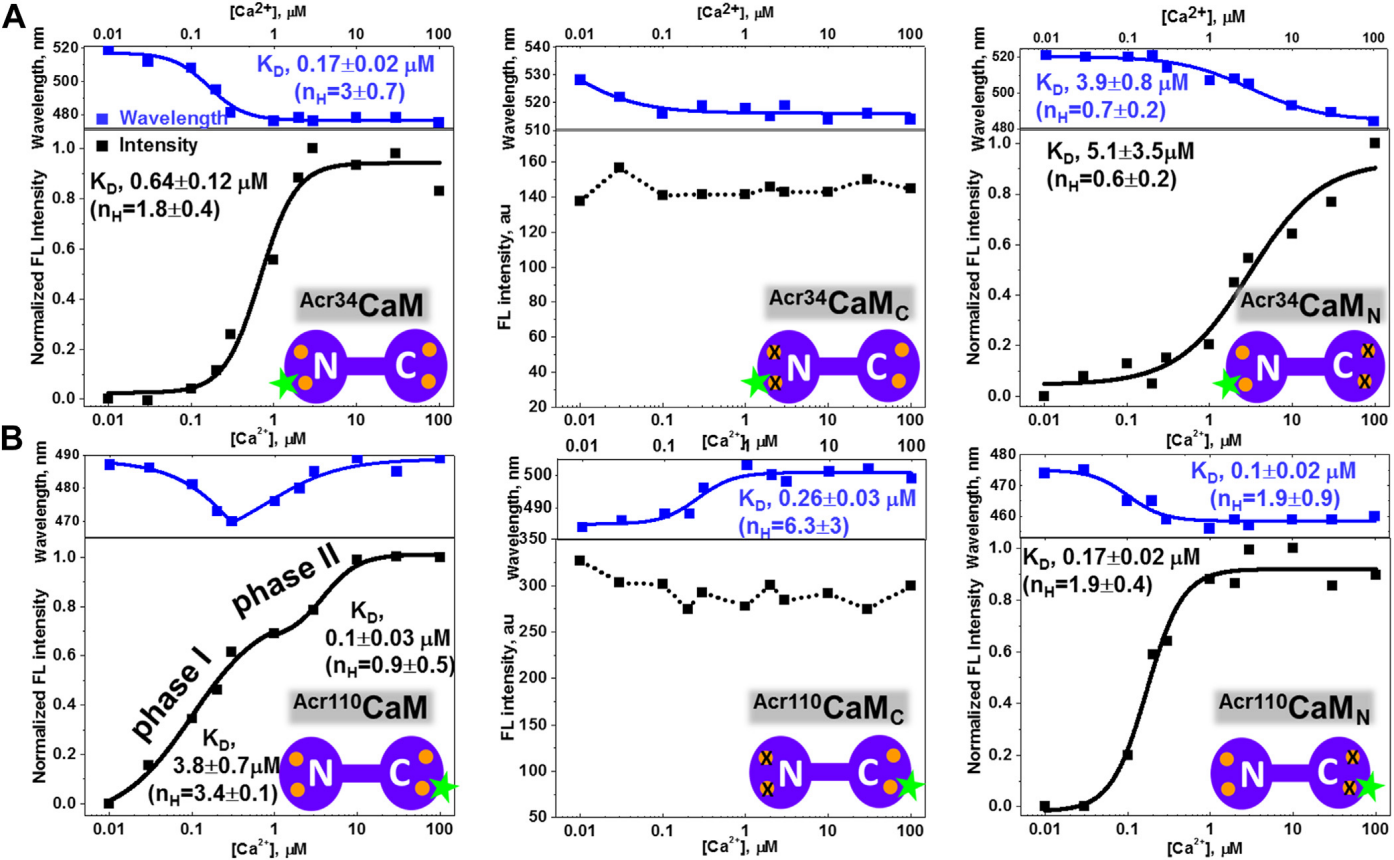
**Steady-state fluorescence measurement of**^**Acr**^**CaM,**^**Acr**^**CaM**_**C**_, **and**^**Acr**^**CaM**_**N**_**in a solution of 20 mM Mops, 30 mM NaCl (pH 7), containing 0.01 to 100 μM [Ca**^**2+**^**].** Each panel includes cartoon schematics describing the acrylodan-labeled CaM (^Acr^CaM) and its lobe-specific Ca^2+^-sensitive mutants retaining Ca^2+^ binding sites at N-lobe (^Acr^CaM_N_) or C-lobe (^Acr^CaM_C_), respectively. The fluorophore (*green star*) was attached at positions T34C or T110C as ^Acr34^CaM_x_ and ^Acr110^CaM_x_ accordingly. The *orange circles* illustrate the Ca^2+^ binding sites at each lobe, crossed *orange circles* indicate ablated Ca^2+^ binding sites. *A*, fluorescence responses to [Ca^2+^] of ^Acr34^CaM, ^Acr34^CaM_C_, and ^Acr34^CaM_N_. *B*, fluorescence response to [Ca^2+^] of ^Acr110^CaM, ^Acr110^CaM_C_, and ^Acr110^CaM_N_. The *blue traces* represent the shift in peak-wavelength of the fluorescence spectrum in response to [Ca^2+^]. The *black traces* represent the change in peak-intensity of the fluorescence spectrum in response to [Ca^2+^]. *Solid lines* represent fits using the Hill function. K_D_ values and Hill coefficients are means ± SD of fits of the experimental data to Hill equations (n = 3). Acr, acrylodan; CaM, calmodulin.

## Results and discussion

### Detection of Ca^2+^ binding to free CaM and lobe-specific Ca^2+^-sensitive CaM mutants *via* steady state fluorescence measurements

To determine the lobe-specific Ca^2+^ binding events, point mutations were introduced in the EF-hands of CaM, to selectively ablate Ca^2+^ binding in the N- or C-lobe. As illustrated in the insets of Figure 1, CaM_C_ allows Ca^2+^ binding only at the C-lobe sites, and CaM_N_ allows Ca^2+^ binding only at the N-lobe. Therefore, these mutants should undergo structural changes only due to Ca^2+^ association or dissociation occurring at the respective CaM lobes (11).

As method validation, we measured the steady state fluorescence emission spectra of Acr-labeled CaM (^Acr^CaM) as a function of [Ca^2+^] to determine if Acr fluorescence is sensitive to structural changes occurring in the C- and N-lobe. As shown in Figure 1*A* (left two panels), increasing [Ca^2+^] caused a ∼40-nm blue shift in the fluorescence emission spectra of ^Acr34^CaM (top panel) and an increase in the peak intensity (whole spectra shown in Fig. S1), suggesting that the environment of the probe becomes more hydrophobic upon Ca^2+^ binding. The plot of fluorescence intensity *versus* [Ca^2+^] exhibits a typical binding profile, with an apparent K_D_ of 0.64 μM. Fitting the measurements of wavelength shift *versus* [Ca^2+^] yields a lower apparent K_D_ (0.17 μM) (Fig. 1*A*). The Hill coefficients and apparent K_D_ values were indicated in Figure 1. The whole fluorescence spectra of the labeled CaMs at designed [Ca^2+^] are shown in Fig. S1.

With ^Acr34^CaM_C_, spectral changes differed from those of ^Acr34^CaM, with much smaller responses observed in peak intensity and blue shift in the peak wavelength (∼14 nm). The wavelength shift is completed between 0.01 and 0.1 μM Ca^2+^. This is consistent with a high affinity for Ca^2+^, suggesting a K_D_ in the low nM range or lower for the ^Acr34^CaM_C_ construct (Fig. 1*A*, middle panel). These small changes in the fluorescence spectra suggested that either (a) the probe attached at the N-lobe (residue 34) is insensitive to the structural changes occurring in the C-lobe, or (b) only subtle structural changes occur in ^Acr34^CaM_C_ upon Ca^2+^ binding to the C-lobe.

With ^Acr34^CaM_N_, the spectral blue shift (∼40 nm) and intensity increase were very similar to those of ^Acr34^CaM, but with a higher apparent K_D_ (3.9 μM from the wavelength plot and 5.1 μM from the intensity plot) (Fig. 1*A*, right panel). Since the apparent K_D_ of ^Acr34^CaM is the combination of Ca^2+^ affinities of both lobes, the larger K_D_ of ^Acr34^CaM_N_ suggests the Ca^2+^ affinity of the N-lobe is lower than the C-lobe, which is consistent with the literature determinations *via* other methods (3).

In contrast to ^Acr34^CaM (probe at the N-lobe), the spectral peak for ^Acr110^CaM (probe at the C-lobe) has a biphasic response to increasing [Ca^2+^]: the emission spectrum peak of ^Acr110^CaM blue shifted ∼17 nm below 0.1 μM Ca^2+^, then red shifted ∼18 nm before plateauing above 10 μM Ca^2+^. This is consistent with two concurrent Ca^2+^-binding processes being detected, with K_D_s of ∼0.6 and ∼0.8 μM based on the fitting (Fig. 1*B*, left, top panel). The intensity change *versus* [Ca^2+^] also appears to be biphasic and can be better fitted with two apparent K_D_ values of ∼0.1 μM and 3.8 μM corresponding to 0.01 to 1 μM and 2 to 100 μM of [Ca^2+^], respectively (Fig. 1*B*, left, bottom panel). For all data sets, the K_D_ values obtained from the peak wavelength data trend smaller than the K_D_ values obtained from the peak intensity data. This is likely due to contributions from minor features of the fluorescence spectra. We are focused on the changes, rather than the absolute values.

With ^Acr110^CaM_C,_ the spectrum red shifted (∼18 nm) in response to [Ca^2+^], but the peak intensity just slightly decreased with the [Ca^2+^] increase (Fig. 1*B*, middle panel). Based on the spectral changes of ^Acr110^CaM_C_, we thus can assign the red shift in ^Acr110^CaM to a structural change in the C-lobe, and the blue shift to a structural change in the N-lobe. The opposite shifts of the peak wavelengths suggest that the environment of the probe at residue 110 becomes more hydrophilic upon Ca^2+^ binding to the C-lobe, while becoming more hydrophobic upon Ca^2+^ binding to the N-lobe. We also observed a much smaller fluorescence response in peak intensity in ^Acr110^CaM_C_, which is in line with ^Acr34^CaM_C_ (Fig. 1*A*, middle panel), corroborating on the interpretation that relatively small structural changes occur in the C-lobe upon Ca^2+^ binding.

With ^Acr110^CaM_N_, we observe a [Ca^2+^]-dependent spectral blue shift (∼17 nm, K_D_ ≈ 0.1 μM) and an intensity increase (K_D_ ≈ 0.17 μM). These K_D_ values are much lower than those from ^Acr110^CaM. This unexpected high Ca^2+^ affinity of ^Acr110^CaM_N_ suggests that Acr-labeling at residue 110 perturbs the local structure of CaM in the Ca^2+^ ablated C-lobe and leads to enhanced Ca^2+^ binding at the N-lobe.

Collectively, the Ca^2+^ titration measurements of steady-state fluorescence from probes at each lobe of CaM indicate that the N- and C- lobes are highly cooperative in Ca^2+^ binding. This is especially the case for the C-lobe, with a fluorescence profile unresponsive to [Ca^2+^] when the N-lobe Ca^2+^ binding is ablated. In alignment with previous structural studies, the change in fluorescence peak intensity and peak wavelength *versus* [Ca^2+^] in one lobe is detected by the probe attached to the other lobe (34, 35, 36, 37). To further validate our fluorescently labeled CaMs, we also investigated overall secondary structure using CD. CD spectra suggest that mutations and labeling did not induce major structural changes in most CaM variants (Fig. S2), except for ^Acr110^CaM_C_, which exhibited a more α helical structure compared to ^Acr110^CaM as indicated by the ellipticity ratio of θ_208_/θ_222_ (0.89 versus 1.0, Fig. S2*e*).

The steady-state measurements suggest that Ca^2+^ binding to CaM results in a larger structural change in the N-lobe compared to the C-lobe (small changes in fluorescence intensity both in ^Acr34^CaM_C_ and ^Acr110^CaM_C,_ middle panels in Fig. 1, *A* and *B*) (38, 39, 40). An alternative explanation for the small changes observed in steady-state fluorescence for the C-lobe is that most of the structural change in that domain relaxes rapidly to the equilibrium state. This points to the importance of understanding the transient kinetics of Ca^2+^ binding to CaM and its lobe-specific Ca^2+^-sensitive CaM variants as used in the steady-state studies above, as discussed below. In addition, results reported here and previously show that the apparent K_D_ values obtained from steady-state fluorescence measurement are highly dependent on the probe and its location (as summarized in Table S1), probably because different fluorescence probes have different sensitivities to the local environment. This is most prominent with ^Acr110^CaM, as we resolve two separate structural changes of CaM upon Ca^2+^ binding (Fig. 1*B*), which was not observed with other probes attached at the same location (13).

### Transient kinetics of Ca^2+^ dissociation from free CaM

To further understand the conformational changes of CaM upon Ca^2+^ binding, we used stopped-flow fluorescence spectroscopy to observe the transient kinetics of Ca^2+^ association/dissociation to/from CaM. Before conducting the kinetics measurements, we performed [^3^H]ryanodine binding assays to evaluate the effect of Acr-labeling on CaM regulation of the RyR1 function. As shown in Fig. S3, the activities of WT-CaM, T34C-CaM, and ^ACR34^CaM in RyR1 regulation are similar both in low and high [Ca^2+^], retaining the typical pattern of RyR1 activation and inhibition, respectively. Although RyR1 regulation is altered for the lobe-specific Ca^2+^-sensitive mutants (CaMc and CaM_N_), labeling has only subtle effects on their activities.

**Figure 2:**
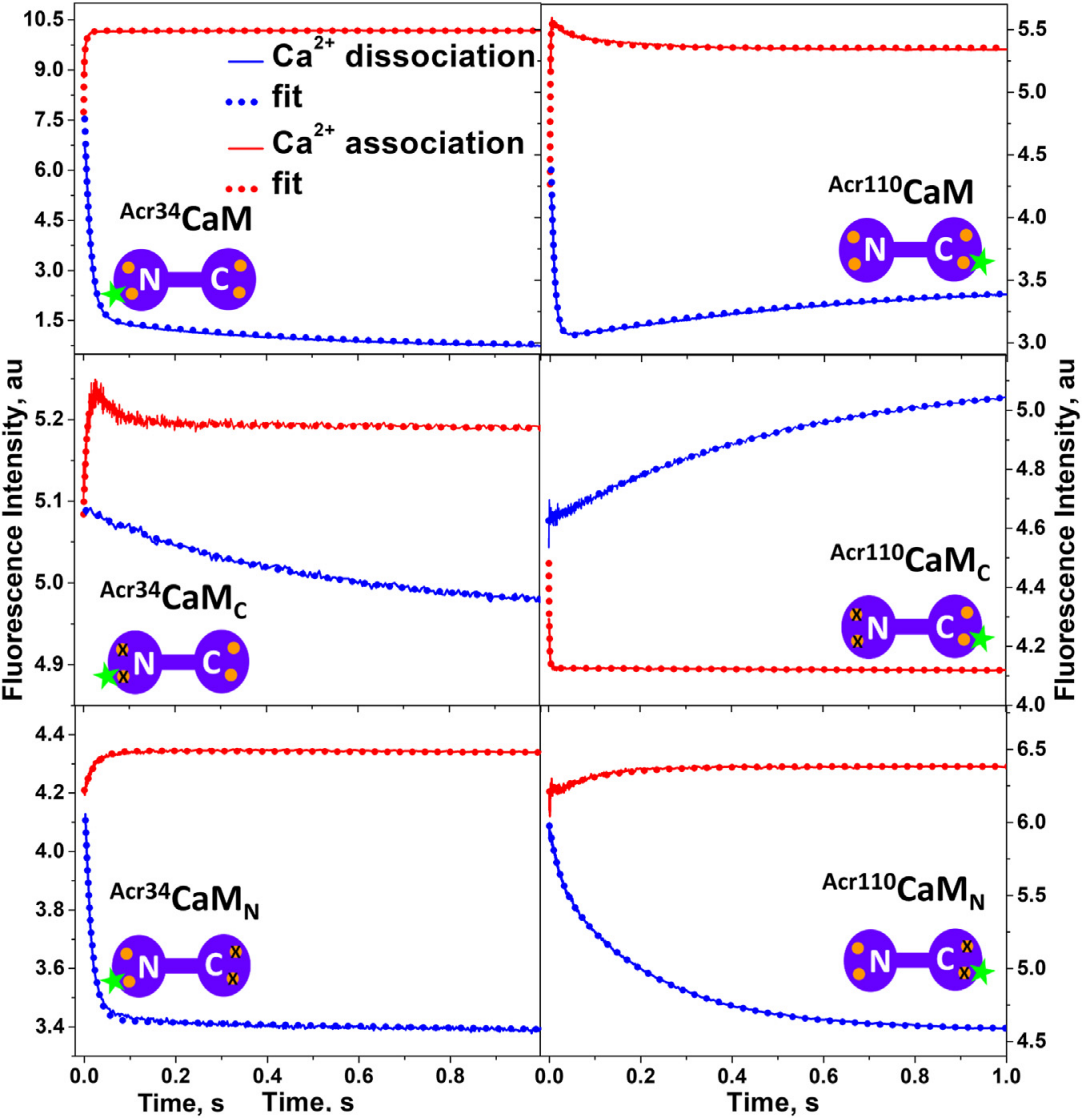
**Transient kinetics of Ca**^**2+**^**binding to free CaM and its lobe-specific Ca**^**2+**^**sensitive mutants CaM**_**N**_**and CaM**_**C**_. Ca^2+^ dissociation (*blue curves*) and association (*red curves*) with acrylodan attached at residue 34 (*left* column) or 110 (*right* column), respectively. *Blue curves*: CaM (0.5 μM) presaturated with 30 μM Ca^2+^ was rapidly mixed with an equal volume of 2 mM EGTA in a stopped-flow apparatus, and the fluorescence change was monitored at 4 °C. *Red curves*: CaM (0.5 μM) was rapidly mixed with an equal volume of 20 μM free Ca^2+^ (EGTA buffered) at 4 °C in 20 mM Mops, 30 mM NaCl, pH 7. The data were fitted with one- or two-exponential functions using Pro-Data Software Suite (version 4.2.12, Applied Photophysics) (*dotted lines*), and fitting results were summarized in Table 1. CaM, calmodulin.

In a stopped-flow apparatus, Ca^2+^-saturated ^Acr^CaM was mixed rapidly with the Ca^2+^ chelator EGTA, and the change in fluorescence of ^Acr^CaM was monitored at 4 °C. Blue traces in Figure 2 represent the time-course of Ca^2+^ dissociation from CaMs with probe attached at the N-lobe or C-lobe (^Acr34^CaM_X_, left column; ^Acr110^CaM_X_, right column). The fluorescence of ^Acr34^CaM, ^Acr34^CaM_C,_ and ^Acr34^CaM_N_ all decreased upon Ca^2+^ dissociation, suggesting that Ca^2+^ dissociation from the N- and C-lobes both make the environment of the probe at residue 34 more hydrophilic, consistent with the observations in steady-state measurements (Fig. 1*A*) (23, 25). The Ca^2+^ dissociation rate constants (Ca^2+^ off-rates) summarized in Table 1 were obtained by fitting these traces with multiexponential functions. A representative fitting and residual are shown in Fig. S4. The primary rate constants of Ca^2+^ dissociation from ^Acr34^CaM_C_ (2.1 s^−1^) and ^Acr34^CaM_N_ (67 s^−1^) matched approximately the two rate constants obtained from ^Acr34^CaM (2 and 76 s^−1^), suggesting that the slow Ca^2+^ dissociation from ^Acr34^CaM occurs at the C-lobe, while the fast dissociation occurs at the N-lobe (21, 23, 25). These rate constants also reveal that Ca^2+^ dissociation from the N-lobe is > 30-fold faster than from the C-lobe. Control experiments with free dye and labeled CaM_1234_ (in which the Ca^2+^ binding sites in both two lobes are ablated) were carried out to ascertain that the change in fluorescence we observed is caused by Ca^2+^ binding to CaM and not the dye itself (Fig. S5) (41).

**Table 1:** Rate constants of the Ca^2+^ dissociation and association to CaM, CaM_C_, and CaM_N_, obtained from the exponential function fit of the time course of kinetics in Figure 3

*k*, s^−1^ (fraction)	CaM				C-lobe			N-lobe			
	^Acr34^CaM		^Acr110^CaM		^Acr34^CaM_C_		^Acr110^CaM_C_	^Acr34^CaM_N_		^Acr110^CaM_N_	
	*k*_1_	*k*_2_	*k*_1_	*k*_2_	*k*_1_	*k*_2_	*k*_1_	*k*_1_	*k*_2_	*k*_1_	*k*_2_
Ca^2+^ dissociation	76 ± 3 (89%)	2.3 ± 0.1 (11%)	109 ± 6 (80%)	1.8 ± 0.3 (20%)	2.4 ± 0.1 (62.5%)	0.13 ± 0.07 (37.5%)	2. 2 ± 0.3 (100%)	67 ± 2 (93%)	0.6 ± 0.02 (7%)	55 ± 7 (41%)	4.7 ± 0.2 (59%)
Ca^2+^ association	1191 ± 33 (91%)	66 ± 1 (9%)	1014 ± 249 (93%)	24.9 ± 1.5 (7%)	100 ± 6 (97%)	20.8 ± 2.5 (3%)	787 ± 18 (100%)	44 ± 1.8 (76%)	0.3 ± 0.03 (24%)	9.9 ± 0.1 (100%)	

The rate constants are shown as mean ± SD (n ≥ 4).

The kinetics results from ^Acr34^CaM are further supported by measurements with the probe attached at the C-lobe, ^Acr110^CaM (blue traces in right column, Fig. 2. Unlike ^Acr34^CaM, Ca^2+^ dissociation from ^Acr110^CaM led to fluorescence change in two distinct phases: an initial rapid fluorescence decrease followed by a slow increase. Each of the two phases may reflect Ca^2+^ dissociation from each lobe. Ca^2+^ dissociation from ^Acr110^CaM_C_ and ^Acr110^CaM_N_ (Fig. 2) further clarifies the assignment: the fluorescence increase corresponds to Ca^2+^ dissociation from the C-lobe, and the fluorescence decrease is the result of Ca^2+^ dissociation from the N-lobe. Ca^2+^ dissociation from the C-lobe, different from the N-lobe, results in a more hydrophobic microscopic environment of the probe at the residue 110. This observation is consistent with the steady-state results, where the spectra of ^Acr110^CaM_C_ red shifted and the intensity decreased upon Ca^2+^ binding, which were opposite to the changes in ^Acr110^CaM_N_ (Fig. 1*B*). As in the case of ^Acr34^CaM, Ca^2+^ dissociation from ^Acr110^CaM was best fitted with two-exponential function (∼109 and 1.8 s^−1^). The Ca^2+^ dissociation rate constant of the C-lobe, obtained from ^Acr110^CaM and ^Acr110^CaM_C_ were similar (1.8 *versus* 2.2 s^−1^), while for N-lobe they were quite different (109 *versus* 55 s^−1^). The difference in dissociation rates between ^Acr110^CaM and ^Acr110^CaM_N_ confirms that a structural perturbation by the labeling at residue 110 affects Ca^2+^ binding at the N-lobe significantly, as observed in the steady-state measurements (Fig. 1*B*). The values of Ca^2+^ dissociation rate of ^Acr110^CaM_X_ may not be reliable due to the labeling interruption at residue 110, but it helps us to assign the phases to each lobe in the kinetics of intact CaM.

The slow Ca^2+^ dissociation rate and small amplitude from the C-lobe in transient kinetics, regardless of the location of probe, indicate that Ca^2+^ dissociation from the C-lobe induces a slow and small structural change. On the other hand, fast dissociation rate and large amplitude from the N-lobe indicates that Ca^2+^ dissociation causes a fast and large structural change on the N-lobe. In addition, the results with ^Acr110^CaM also confirm the findings obtained from ^Acr34^CaM that Ca^2+^ dissociation from the N-lobe of CaM is faster than that from the C-lobe, which is consistent with the previous observations using N-lobe or C-lobe CaM fragments (42). These results convinced us that we can characterize the structural changes of the two lobes in response to Ca^2+^ dissociation, through monitoring the kinetics of intact CaM.

To compare with previously reported Ca^2+^ off-rates that had been determined by a variety of techniques at different temperatures (21, 22, 23, 25, 26, 27), we have measured Ca^2+^ dissociation at temperatures ranging from 4 to 20 °C. The ln*k*_1_ (primary rate constant) *versus* temperature plots were linearly extrapolated to the previously reported room temperature (25 °C). As compared in Table S2, the results obtained using our lobe-specific fluorescent CaMs are consistent with the data obtained *via* ^1^H- and ^43^Ca-NMR. This result further confirms that our approach using fluorescently labeled CaM is appropriate for the study of the kinetics of Ca^2+^ dissociation from CaM free in solution or in complex with RyR1.

### Transient kinetics of Ca^2+^ association to free CaM

Ca^2+^ association to CaM and lobe-specific Ca^2+^-sensitive constructs, CaM_C_ and CaM_N_, were measured by rapidly mixing ^Acr^CaM_X_ with 20 μM free [Ca^2+^] solution and monitoring with a stopped-flow instrument. Red traces in Figure 2 are time courses of fluorescence change of ^Acr34^CaM_X_ (left column) and ^Acr110^CaM_X_ (right column). The fluorescence intensity of ^Acr34^CaM, ^Acr34^CaM_C,_ and ^Acr34^CaM_N_ increased with time upon Ca^2+^ binding, inversely corresponding to Ca^2+^ dissociation (the blue trace in each panel), suggesting that Ca^2+^ binding induced a more hydrophobic environment for the probe. The rate constants of Ca^2+^ association obtained by fitting the red traces with exponential functions are summarized in Table 1. ^Acr34^CaM exhibited very rapid Ca^2+^ association rate constants (1191 and 65 s^−1^), which are faster than those dominant rate constant of ^Acr34^CaM_C_ (100 s^−1^) and ^Acr34^CaM_N_ (44 s^−1^). Part of the initial fast phase of ^Acr34^CaM took place in the dead time of the instrument (assuming it is a first-order reaction, t_1/2_ corresponding to *k*_1_ of 1191 s^−1^ is 0.8 ms, which is shorter than the instrument dead time of 1.5 ms), the fitting was confirmed by calibrating the dead time of the instrument as shown in Fig. S6 (43). The signal amplitudes of Ca^2+^ association to ^Acr34^CaM_N_ and to ^Acr34^CaM_C_ are only ∼11% and ∼5% of the ^Acr34^CaM, respectively (Fig. S7*A*), which is consistent with the signal amplitudes of Ca^2+^ dissociation from ^Acr34^CaM_X_ (Fig. S7*B*, signal amplitudes of ^Acr34^CaM_N_ and ^Acr34^CaM_C_ are ∼12% and ∼2% of intact ^Acr34^CaM, respectively). These results suggest that Ca^2+^ binding elicits a larger structural change in intact CaM than in the lobe-specific Ca^2+^-sensitive CaMs. That is, cross-talk between the two lobes of CaM in Ca^2+^ binding results in a larger structural change (34, 35, 36, 37, 44). Even though the fluorescence changes of Ca^2+^ association/dissociation of lobe-specific Ca^2+^-sensitive mutants are smaller than the intact CaM, it is still clear that Ca^2+^ binding to ^Acr34^CaM_C_ is at least two-fold faster than to ^Acr34^CaM_N_. Therefore, the fast phase in the Ca^2+^ association kinetics of the intact CaM can be assigned to the C-lobe, and the slow phase to the N-lobe. Although the structural status of the lobe-specific Ca^2+^-sensitive CaM mutants may not be identical to the intact CaM, they are comparable, as suggested by the similar Ca^2+^ dissociation rates of each lobe.

In contrast to ^Acr34^CaM, Ca^2+^ association to ^Acr110^CaM is complex. Two opposite phases of fluorescence change were observed in ^Acr110^CaM upon Ca^2+^ association (Fig. 2, red trace in the top panel of the right column): a fast increase (∼1000 s^−1^) and a slow decrease (25 s^−1^). A fast fluorescence decrease (∼787 s^−1^) was observed in ^Acr110^CaM_C_ (Fig. 2 red trace in the middle panel of the right column), with a small amplitude. A slow increase (∼10 s^−1^) was observed in ^Acr110^CaM_N_ upon Ca^2+^ association (Fig. 2 red trace in the bottom panel of the right column). It appears that the change in fluorescence of ^Acr110^CaM is not a simple summation of the changes of ^Acr110^CaM_N_ and ^Acr110^CaM_C_, in terms of the amplitude and direction of the signal change. This further supports the notion that Ca^2+^ binding to the two lobes of CaM is highly cooperative, as suggested by Ca^2+^ binding kinetics with ^Acr34^CaM and previous studies (34, 35, 36, 37, 44). It is worthy to note that the complexity of the kinetics data of Ca^2+^ association to ^Acr110^CaM further suggests that acrylodan labeling at position 110 of CaM significantly disrupts Ca^2+^ binding to CaM.

To summarize our conclusions from Ca^2+^ binding kinetics to free CaM_X_ (Fig. 2), the kinetics of Ca^2+^ dissociation/association from/to CaM identify unambiguously the structural changes of each lobe. The kinetics of Ca^2+^ dissociation from CaM and lobe-specific Ca^2+^-sensitive CaMs with the fluorescent probe at either lobe of CaM, show that Ca^2+^ dissociation from the N-lobe of CaM is ∼30-fold faster than that from the C-lobe. In Ca^2+^ association, even though the discrepancy in rate constants between the intact CaM and lobe-specific Ca^2+^-sensitive CaMs is significant (especially for ^Acr110^CaM), it is clear that Ca^2+^ association to the C-lobe is faster than to the N-lobe (Table 1). Previous research suggested the order of the Ca^2+^ affinity of the four Ca^2+^ binding sites are III, IV (C-lobe) > II, I (N-lobe) (42, 45), but the order of Ca^2+^ association rates was controversial (26). For practical reasons (instrument dead-time), we cannot determine Ca^2+^ association rates directly from the current kinetic data, but under the same conditions, larger (faster) Ca^2+^ association rate constant of the C-lobe indicates that the Ca^2+^ association rate of the C-lobe is faster than that of the N-lobe (27).

### Kinetics of Ca^2+^ binding to the CaM/RyR1 complex

**Figure 3:**
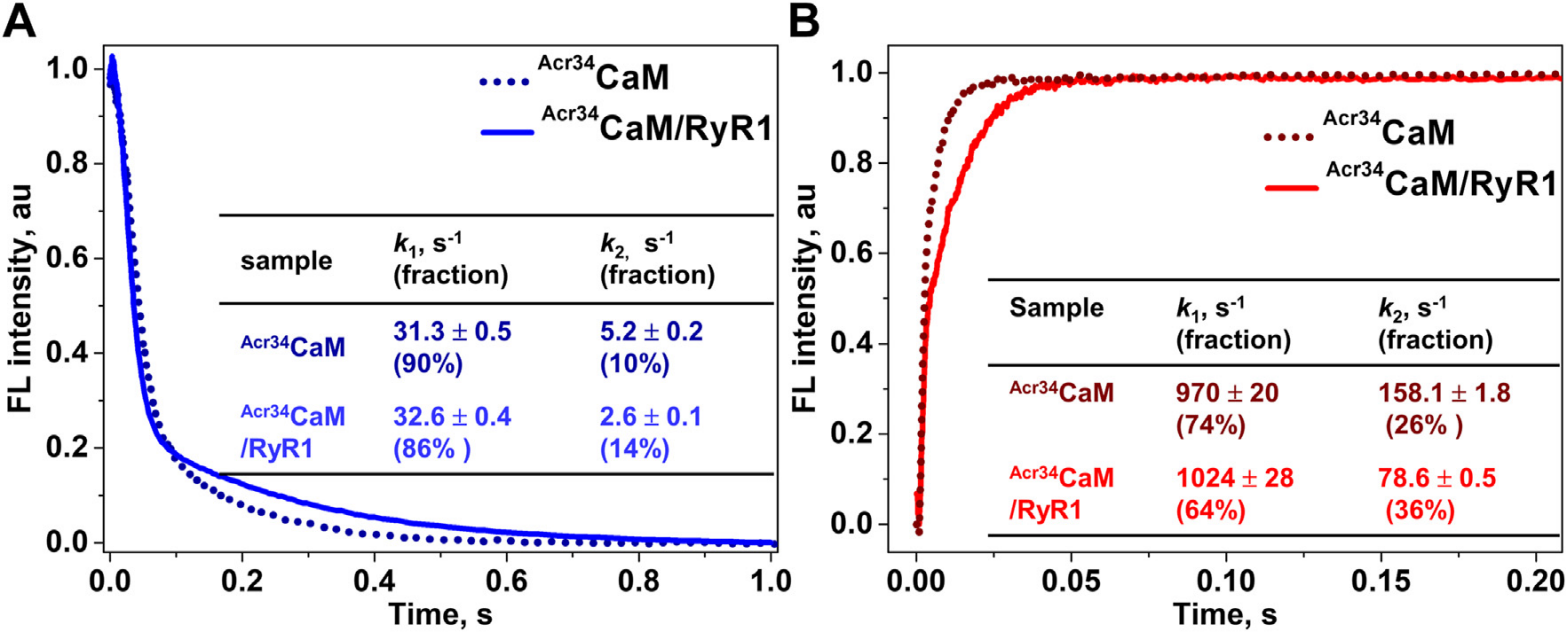
**Kinetics of Ca^2+^ binding to the CaM/RyR1 complex.***A*, Ca^2+^ dissociation from ^Acr34^CaM in the absence (*dotted curves*) and presence of excess purified RyR1 (*solid curves*). *B*, Ca^2+^ association to ^Acr34^CaM in the absence (*dotted curves*) and presence of excess purified RyR1 (*solid curves*). The experiments were performed as described in Figure 2, except for the 2 h incubation of CaM with RyR1, in a solution containing 20 mM Pipes, 150 mM KCl, 5 mM GSH, 1 μg/ml of aprotinin/leupeptin, and 2 mM DTT, pH 7. The final concentration of RyR1-CaM binding sites was ∼2.5-fold of the added [CaM]. Fitting results are shown as means ± SD (n = 3) and summarized in the corresponding inset tables. Acr, acrylodan; CaM, calmodulin; RyR, ryanodine receptor.

The kinetics of Ca^2+^ dissociation and association of free CaM established a technical foundation for the study of Ca^2+^ binding to CaM when in complex with RyR1, as needed to understand the mechanism of Ca^2+^-driven RyR1 regulation by CaM. Before mixing with the Ca^2+^ chelator EGTA, labeled CaM was incubated with excess purified RyR1 to ensure that all CaM were in the RyR1-bound state (per Experimental procedures). Considering the structural perturbation that labeling at residue 110 might cause (based on the results with free ^Acr110^CaM), we chose to focus on ^Acr34^CaM/RyR1. Figure 3 displays time courses of fluorescence change for ^Acr34^CaM/RyR1 upon Ca^2+^ dissociation (Fig. 3*A*) and association (Fig. 3*B*), overlaid on the respective free-CaM measurements under the same conditions. As in the case of free ^Acr34^CaM, fluorescence of ^Acr34^CaM/RyR1 decreases upon Ca^2+^ dissociation from the complex, and the fluorescence change is faster for the N-lobe than the C-lobe (Fig. 3*A*). However, compared to free ^Acr34^CaM, the dissociation rate constant from the N-lobe (*k*_1_) is similar, while the dissociation rate constant from the C-lobe (*k*_2_) of ^Acr34^CaM/RyR1 is two-fold slower (Fig. 3*A*, inset Table). The changes in the rate constants of Ca^2+^ dissociation imply that Ca^2+^ dissociation from the C-lobe of CaM causes a slower structural change after RyR1 binding, while the structural change caused by Ca^2+^ dissociation from the N-lobe is virtually unaffected by RyR1 binding.

Our measurement of Ca^2+^ association to CaM in complex with RyR1 (^Acr34^CaM/RyR1) revealed that the initial fast fluorescence change of the C-lobe (*k*_1_) was affected insignificantly, but the association rate constant of the N-lobe (*k*_2_) is ∼ two-fold slower than free ^Acr34^CaM (Fig. 3*B*).

Compared to free CaM, the results of Ca^2+^ association together with the Ca^2+^ dissociation from ^Acr34^CaM/RyR1, suggest that Ca^2+^ affinity for the C-lobe of CaM increases, while Ca^2+^ affinity for the N-lobe decreases by RyR1 binding. Nevertheless, the averages of these rate constants indicate a negligible effect of purified RyR1 on the overall CaM Ca^2+^ affinity. In our previous report using steady-state fluorescence measurements to compare Ca^2+^ binding to free and RyR1-bound CaM, we observed a ∼3-fold increase in overall Ca^2+^ affinity for RyR1-bound CaM (11). This discrepancy could be due to using HSR samples in our previous study *versus* purified RyR1 in this case, which is devoid of potentially confounding CaM binding targets.

**Figure 4:**
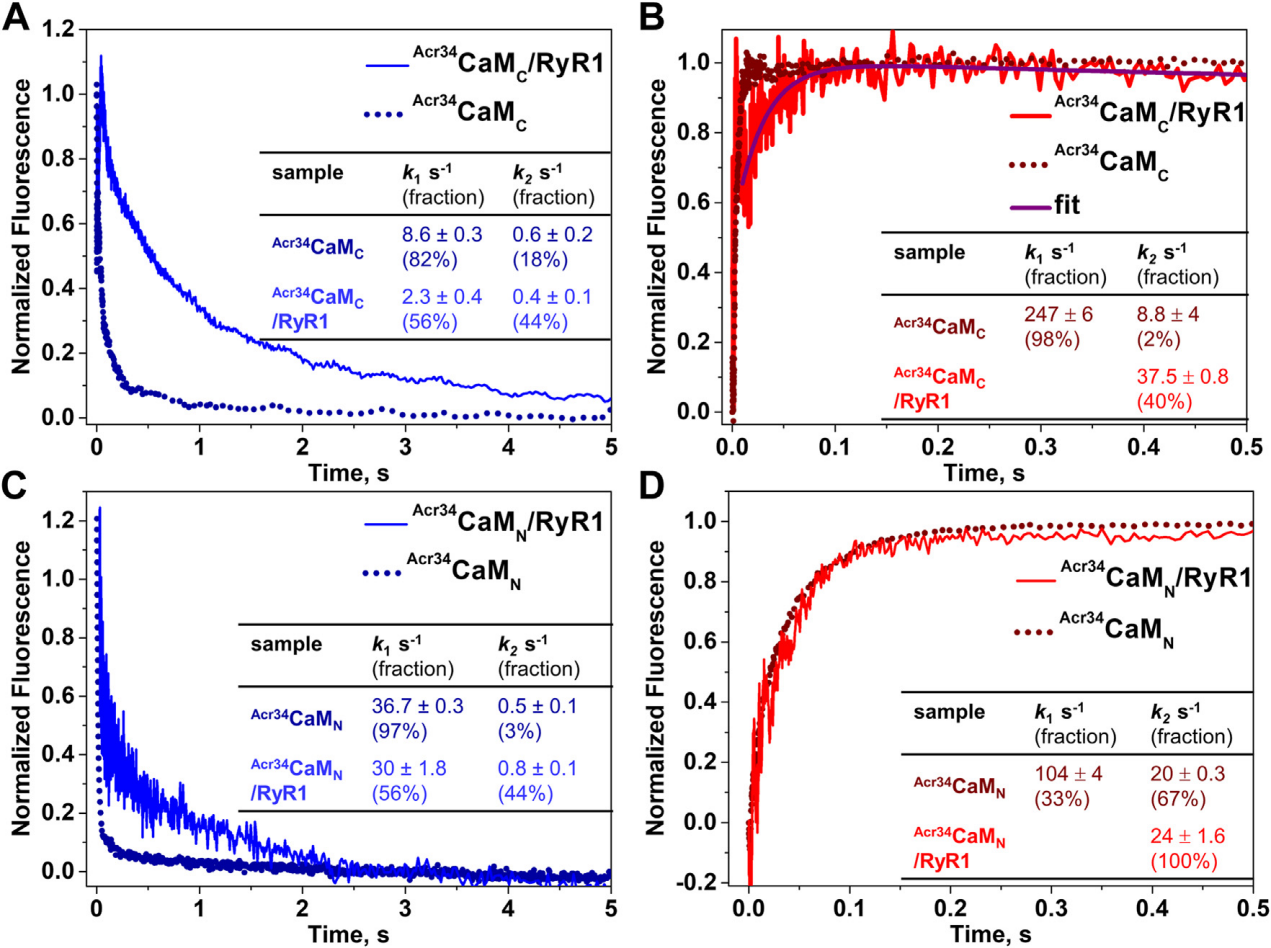
**Kinetics of Ca**^**2+**^**binding to CaM**_**C**_**and CaM**_**N**_**in complex with RyR1.** Ca^2+^ dissociation/association to ^Acr34^CaM_C_ (*A* and *B*) and ^Acr34^CaM_N_ (*C* and *D*) in the absence (*dotted curves*) and presence (*solid curves*) of purified RyR1. Experimental conditions and data analysis are the same as in Figure 3. Fitting results are shown as means ± SD (n = 3) and summarized in the corresponding inset tables. Acr, acrylodan; CaM, calmodulin; RyR, ryanodine receptor.

To further evaluate this finding, we examined the kinetics of Ca^2+^ dissociation and association using lobe-specific Ca^2+^ sensitive CaM constructs in complex with RyR1–^Acr34^CaM_C_/RyR1 and ^Acr34^CaM_N_/RyR1 (Fig. 4). Figure 4*A* shows that Ca^2+^ dissociation from ^Acr34^CaM_C_/RyR1 (blue trace) was three-fold slower than from free ^Acr34^CaM_C_ (dark blue dotted trace), which is in line with the observation in the intact ^Acr34^CaM/RyR1 (Fig. 3*A*). Figure 4*B* compares Ca^2+^ association to ^Acr34^CaM_C_ in the presence *versus* absence of RyR1. The fast-increase phase that we observed with free ^Acr34^CaM_C_ was not resolved with the ^Acr34^CaM_C/_RyR1 complex due to low S/N (data can be fitted reliably only beyond 15 ms, when the fast phase of ^Acr34^CaM_C_ is already complete). We infer that this unresolved initial phase could be at least comparable or even faster than that of the free CaM_C_ based on the starting of the signal. Instead, we observed a much slower phase with an amplitude that is only about one-quarter of the total signal of free CaM_C_. We therefore speculate that the observed slow phase of Ca^2+^ association to the RyR1-bound CaM_C_ could be contributed by one Ca^2+^ binding site of the C-lobe. Combining Figure 4, *A* and *B*, we conclude that the Ca^2+^ affinity of at least one of the Ca^2+^ binding sites of the C-lobe of CaM was affected by RyR1 binding, supporting partially the conclusion with the intact CaM/RyR1 (Fig. 3).

**Figure 5:**
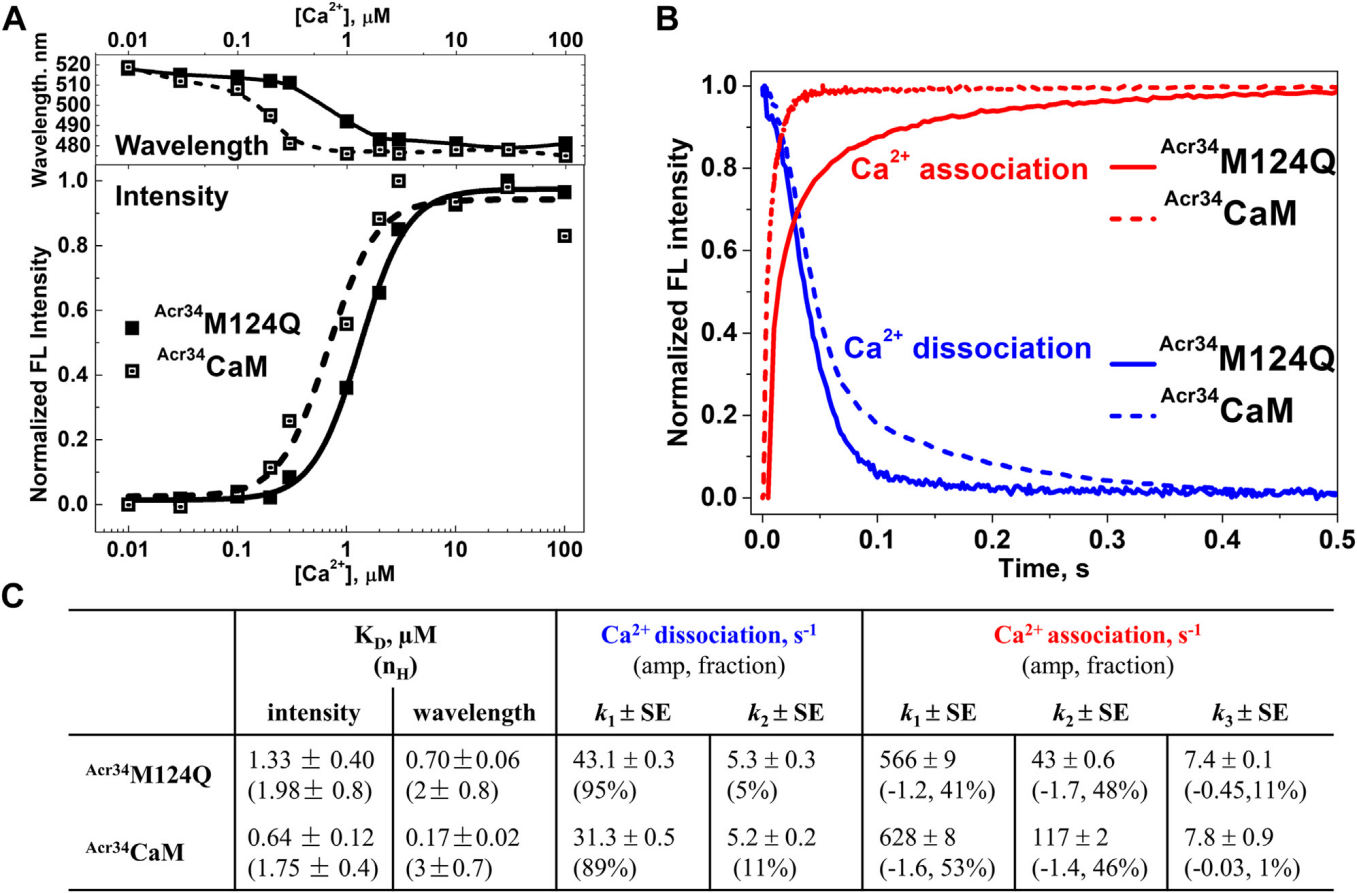
**Ca**^**2+**^**binding to oximimetic CaM mutant M124Q free in solution*****.**A*, steady-state fluorescence of ^Acr34^M124Q. *Top panel*: fluorescence peak-wavelength *versus* [Ca^2+^]. *Bottom panel*: peak-intensity *versus* [Ca^2+^], as described in Figure 1. *Open squares* represent ^Acr34^CaM, and *closed squares* represent ^Acr34^M124Q. *Solid lines* are fits to the Hill equation. *B*, time courses of Ca^2+^ binding events of ^Acr34^M124Q (*solid traces*) and ^Acr34^CaM (*dotted traces*) under the same conditions as described in Experimental procedures. *C*, Ca^2+^ affinity (K_D_) and rate constants of Ca^2+^ association/dissociation from ^Acr34^CaM and ^Acr34^M124Q obtained from (*A*) and (*B*), respectively. K_D_ values are fits of the experiments to the Hill equation, mean ± SD (n = 3). Rate constants are shown as means ± SD (n ≥ 3). Acr, acrylodan; CaM, calmodulin.

Figure 4, *C* and *D* show Ca^2+^ dissociation and association at ^Acr34^CaM_N_/RyR1. In the case of Ca^2+^ dissociation, the rate constants are slightly altered in the ^Acr34^CaM_N_/RyR1 (Fig. 4*C*), which is consistent with the small changes of Ca^2+^ dissociation in ^Acr34^CaM/RyR1 (Fig. 3*A*). Different from free ^Acr34^CaM where one phase is dominant (larger fraction), in ^Acr34^CaM_N_/RyR1, the signal can be better fitted with two phases contributing comparably (56 and 44%) to the whole signal, suggesting that after RyR1 binding, two Ca^2+^ binding sites at the N-lobe of the CaM_N_ are more distinguishable in the RyR1-bound state. Figure 4*D* shows that one ∼5-fold slower phase of Ca^2+^ association is observed in ^Acr34^CaM_N_/RyR1 compared to the free ^Acr34^CaM_N_, consistent with the result from intact ^Acr34^CaM/RyR1 (Fig. 3*B*). Figure 4, *C* and *D* together support the findings with intact ^Acr34^CaM/RyR1 that Ca^2+^ affinity at the N-lobe of CaM is decreased by RyR1 binding. As we discussed earlier, the structures of lobe-specific Ca^2+^-sensitive CaMs might not be identical to WT-CaM (Fig. 2), thus small discrepancies in the rate constants of Ca^2+^ association to both lobes in the lobe-specific CaMs/RyR1 *versus* intact CaM/RyR1 are not surprising. However, the overall changes in rate constants at the two lobes of CaM_X_/RyR1 (Fig. 4) are close to CaM/RyR1 (Fig. 5).

Collectively, the transient kinetics of Ca^2+^ binding to CaM/RyR1 indicates that RyR1 binding does not alter the overall sequence of Ca^2+^ binding to the two lobes of CaM, but it does slow down Ca^2+^ dissociation from the C-lobe and association to the N-lobe (Figs. 3 and 4). The changes in the rate constants of Ca^2+^ association and dissociation eventually result in an enhanced Ca^2+^ affinity at the C-lobe (one Ca^2+^ binding site at least) and decreased Ca^2+^ affinity at the N-lobe in CaM/RyR1. The larger Ca^2+^ association rate constants of CaM/RyR1 *versus* CaM_C_/RyR1 and CaM_N_/RyR1 indicate the retention of cooperativity between the two lobes of CaM when bound to RyR1.

Based on the kinetics of Ca^2+^ binding to CaM/RyR1, during EC coupling, as Ca^2+^ is released from the sarcoplasmic reticulum (SR), RyR1 is activated by the local rise in [Ca^2+^], which will also lead to a rapid Ca^2+^ binding to the C-lobe of CaM, thus causing a fast structural change of CaM to initiate its inhibition of RyR1. The following slower structural change of the N-lobe of CaM upon Ca^2+^ binding further reinforces the closure of RyR1, until Ca^2+^ is pumped back into the SR (by the Ca^2+^-ATPase pumps) and the cytoplasmic [Ca^2+^] returns to the resting level. The N-lobe-mediated RyR1 inhibition is ∼10 times slower than the initial inhibition by the C-lobe, which is sufficiently slow to maintain the RyR1 in an activated form during the initial Ca^2+^ release phase. The ^ACR^CaM kinetics of Ca^2+^ association is faster than the complementary, FRET-based, kinetics measurement of the CaM conformational change (20). This is structurally logical as the Ca^2+^-association must precede the larger scale structural changes observed *via* FRET. The suggested mechanistic model of the N-lobe *versus* C-lobe roles is also supported by the findings by Fruen *et al.* that mutations of CaM’s C-terminal Ca^2+^ sites abolished CaM inhibition to RyR1 (41). When [Ca^2+^]_cyto_ is lowered, slow Ca^2+^ dissociation from CaM compared to fast Ca^2+^ association, provides a delay during which [Ca^2+^]_cyto_ falls sufficiently low before the RyR1 returns to its resting state (6), and CaM shifts back to its original binding site of RyR1 as an activator at nanomolar [Ca^2+^] (46). In this process, the C-lobe is responsible for initiating the inhibition of RyR1 at high [Ca^2+^]_cyto_ by fast Ca^2+^ binding. The N-lobe is necessary for timely reinforcing the inhibition and shifting back to the activation state at low [Ca^2+^] by slow Ca^2+^ association and fast Ca^2+^ dissociation. Thus, both the C- and N-lobe of CaM are necessary for fine tuning this process (13, 41, 47). Recent cryo-EM reports show structural differences of the N- and C-lobes occurring in apo-CaM/RyR *versus* Ca^2+^-CaM/RyR (14, 15, 17). The regulatory mechanism of CaM, could differ from target to target depending on the relative rates of Ca^2+^ association and dissociation to and from the two lobes of CaM (48).

### Kinetics of Ca^2+^ dissociation/association to oximimetic CaM mutant M124Q, free and in complex with RyR1

CaM mutant M124Q has been used to mimic both the structural and functional effects of methionine oxidation on CaM's regulation of RyR1 (49). It has been reported that M124Q decreases the affinity of both apo- and Ca^2+^-CaM for RyR1 (31). Recently, the M124Q mutation was proposed to cause localized unfolding of the CaM C-lobe, preventing the formation of a hydrophobic cluster of residues near the EF-hand Ca^2+^ binding sites (50). However, the dysregulation mechanism remains unknown, whether the M124Q mutation alters Ca^2+^ affinity of CaM or CaM affinity to RyR1 (or both). Therefore, to understand the mechanism of M124Q regulation of RyR1, we have measured the kinetics of Ca^2+^ binding to free M124Q and in M124Q/RyR1 complex.

First, we examined the steady state [Ca^2+^]-dependent fluorescence change of acrylodan attached at residue 34 of M124Q (termed ^Acr34^M124Q). Figure 5*A* shows the change in fluorescence intensity and peak wavelength as a function of [Ca^2+^]. The apparent Ca^2+^ affinity of ^Acr34^M124Q is at least two-fold lower than ^Acr34^CaM (Table in Fig. 5*C*) (31). The Ca^2+^ titration curves of the lobe-specific Ca^2+^-sensitive mutants ^Acr34^M124Q_C_ and ^Acr34^M124Q_N_ (Fig. S8*A*) are very different from those of ^Acr34^CaM_C_ and ^Acr34^CaM_N_. The K_D_ of the ^Acr34^M124Q_C_ can be easily determined, while for ^Acr34^M124Q_N,_ a very small intensity change and wavelength shift are observed, suggesting decreased Ca^2+^ sensitivity of the N-lobe of M124Q. In addition, the Ca^2+^ affinities of ^Acr34^M124Q_N_ and ^Acr34^M124Q are both lower than that for ^Acr34^CaM (Fig. S8*B*) (31).

The Ca^2+^ dependent gel mobility shift of M124Q is smaller *versus* WT CaM (31), also indicating its lower Ca^2+^ sensitivity. Acrylodan labeling of M124Q and its lobe-specific mutants does not affect their gel mobility. We also observe that ^Acr34^M124Q_N_ is less sensitive to Ca^2+^ compared to ^Acr34^M124Q_C_ (Fig. S8*C*) (31). The CD spectra of M124Q *versus* WT-CaM have revealed minor changes by the mutation on the overall structure (50). The CD spectra of T34C/M124Q, T34C/M124Q_C_, and T34C/M124Q_N_ (Fig. S9) are very similar to each other and to their acrylodan-labeled forms, indicating minor overall structural changes by the mutations for ablation of the Ca^2+^ binding sites in M124Q mutant. Together, the steady-state florescence, Ca^2+^ dependent gel mobility, and CD spectra suggest that the M124Q mutation does not dramatically alter the overall structure of CaM, but rather it decreases the Ca^2+^ affinity for the two lobes of CaM (Fig. S8), and the Ca^2+^ affinity to the N-lobe is dramatically affected.

To determine whether the M124Q mutation affects the kinetics of Ca^2+^ binding to free CaM, Ca^2+^ dissociation/association to ^Acr34^M124Q was monitored using the stopped-flow fluorescence and compared with WT-^Acr34^CaM. The directions and amplitudes of fluorescence changes in ^Acr34^M124Q in Ca^2+^ association/dissociation are similar to those of ^Acr34^CaM (Fig. 5*B*), but their rate constants are different. Ca^2+^ dissociation from the N-lobe of ^Acr34^M124Q is faster (43 *versus* 31 s^−1^), while minor change is observed in the C-lobe of ^Acr34^M124Q compared to the WT-^Acr34^CaM (5.3 *versus* 5.2 s^−1^). Rate constant of Ca^2+^ association to the N-lobe of ^Acr34^M124Q was reduced ∼2.5-fold, and C-lobe of ^Acr34^M124Q was slightly reduced compared to WT-^Acr34^CaM (solid red traces in Fig. 5*B*). The increased Ca^2+^ dissociation rate constant and decreased Ca^2+^ association rate constant of the N-lobe suggest that the Ca^2+^ affinity to the N-lobe of M124Q is lower than that of WT-CaM, which is consistent with the overall higher K_D_ from the steady-state measurements (Figs. 5*A* and S8). On the other hand, the Ca^2+^ affinity at the C-lobe of M124Q, which is dramatically decreased in the steady-state measurements with ^Acr34^M124Q_C_ (Fig. S8), is only slightly decreased in transient kinetics with intact M124Q (Fig. 5*B*). This discrepancy between intact and lobe-specific Ca^2+^-sensitive mutants suggests that the microenvironment of the probe in the mutants is different from intact M124Q, though there is no dramatic difference in their CD spectra. Overall, the kinetics results of Ca^2+^ binding to M124Q are consistent with the steady state results.

**Figure 6:**
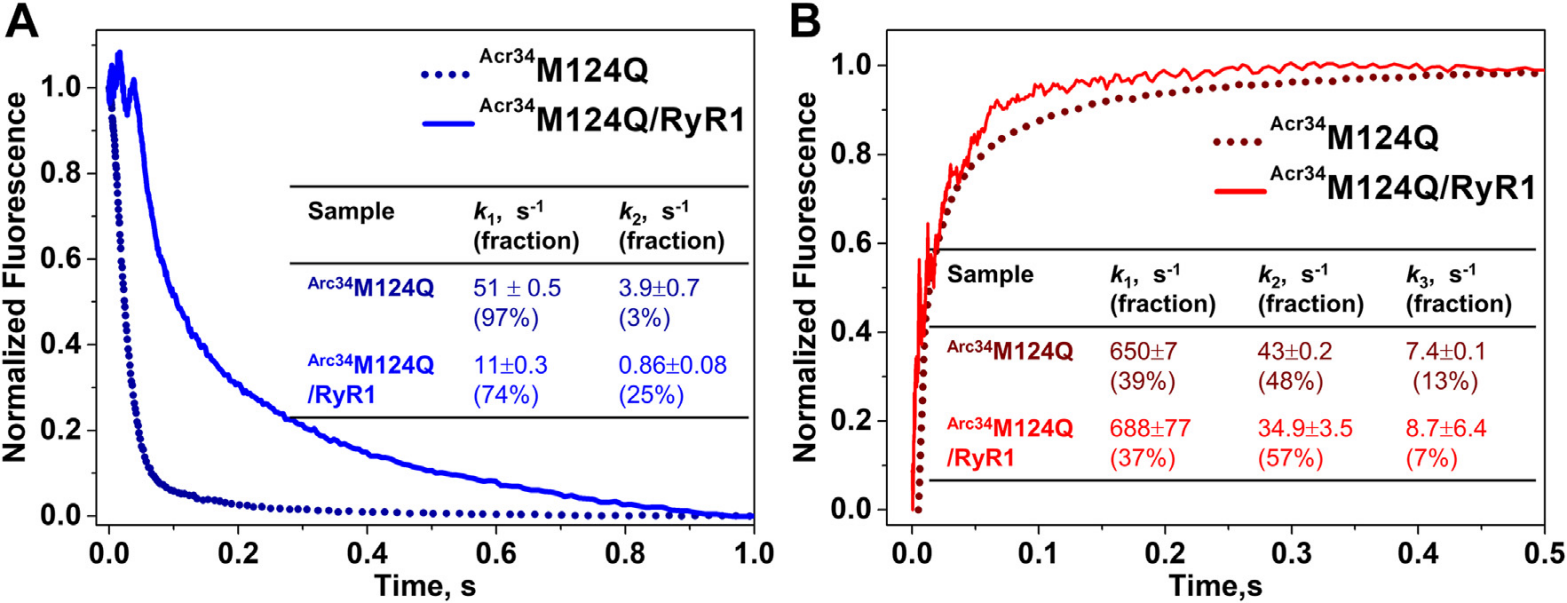
**Kinetics of Ca**^**2+**^**binding to oximimetic CaM mutant M124Q in complex with RyR1.** Kinetics of Ca^2+^ dissociation (*A*) and association (*B*) to ^Acr34^M124Q in the presence of purified RyR1. The *dotted curves* represent the corresponding controls in the absence of RyR1. The experimental conditions and data analysis are the same as in Figure 3. Rate constants are shown as means ± SD (n = 3) and summarized in the corresponding inset tables. Acr, acrylodan; RyR, ryanodine receptor.

For RyR1-bound ^Acr34^M124Q/RyR1, Ca^2+^ dissociation best fits a two-exponential model, as observed above for ^Acr34^CaM/RyR1 (Fig. 3*A*). The Ca^2+^ dissociation rates of C- and N-lobe of ^Acr34^M124Q/RyR1 are both ∼5-fold slower compared to free ^Acr34^M124Q (Fig. 6*A*). The trace of Ca^2+^ association to ^Acr34^M124Q/RyR1 is similar to free ^Acr34^M124Q (Fig. 6*B*). The slower dissociation rates and unaffected association rate constants together indicate that Ca^2+^ affinity at the both lobes of M124Q is increased to the same extent by RyR1 binding.

Steady state fluorescence, gel mobility, and kinetics together suggest that dysregulation of RyR1 by M124Q is due to its intrinsically lower affinity for Ca^2+^ (Figs. 5, S8). Although binding to RyR1 increased M124Q Ca^2+^ affinity, the slower Ca^2+^ association to M124Q, especially at the N-lobe, reduces its sensitivity to the rapid variation of [Ca^2+^] in cells, where it would not interact with RyR1 timely and effectively to regulate its channel function. The lower M124Q affinity for RyR1 may exacerbate this effect (Fig. S10) (31). This is consistent with an earlier FRET study, which proposed that M124Q alters CaM's functional interaction with its target due to a reduced Ca^2+^-dependent structural shift (49).

## Conclusions

**Figure 7:**
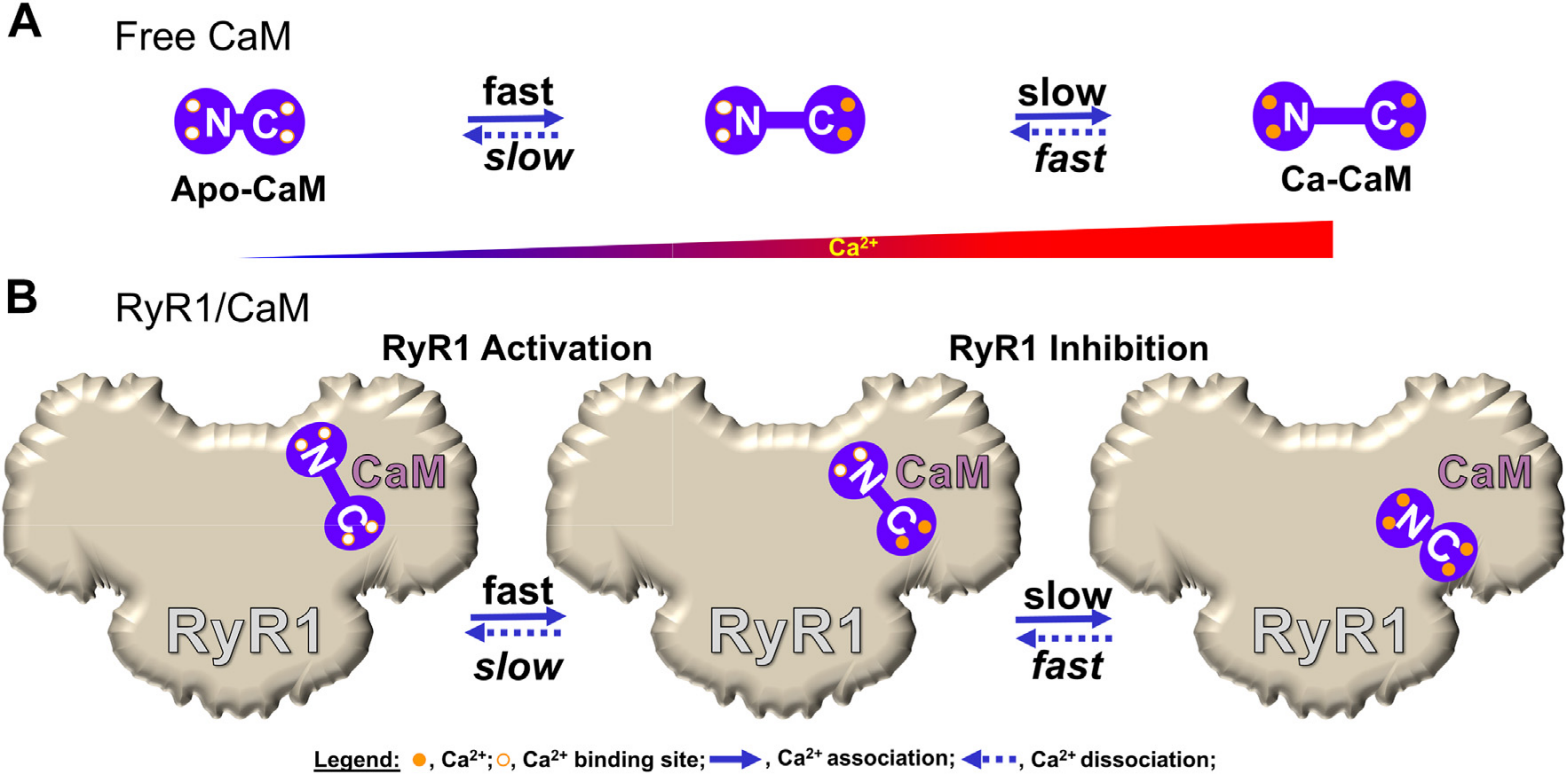
**Model of Ca^2+^ binding to CaM free and in complex with RyR1.***A*, Ca^2+^ association to the C-lobe of free CaM is faster than to the N-lobe, and Ca^2+^ dissociation from the N-lobe CaM is faster than from the C-lobe. Apo-CaM is more compact (*left*) than Ca^2+^-bound CaM (*center* and *right*) (19). *B*, RyR1 binding to CaM does not alter the overall sequence of Ca^2+^ binding to the two lobes of CaM. Fast Ca^2+^ binding to the C-lobe initiates the inhibition of RyR1 at high [Ca^2+^] while the slow Ca^2+^ association to the N-lobe reinforces the timely inhibition. Upon Ca^2+^ return to the basal level, fast Ca^2+^ dissociation from the N-lobe enables the return of CaM to its state of RyR1 activator at low [Ca^2+^]. Two Ca^2+^ binding sites in each domain are indicated in the apo- or calcium-loaded state. The illustrated locations of CaM on RyR are based on cryo-EM structures for apo-CaM/RyR1 (*left*) (15), Ca^2+^-CaM/RyR1 (*center*) (17), whereas the location of Ca^2+^-CaM on the inhibited RyR1 (*right*) reflects FRET-based trilateration results (20) and the Ca^2+^-CaM/RyR2 structure (14). “Fast” and “slow” indicate the rate constants of Ca^2+^-association and dissociation from the CaM. Ca^2+^ association and dissociation from CaM is indicated by *solid* and *dotted arrows*, respectively. CaM, calmodulin; RyR, ryanodine receptor.

We monitored the fluorescence responses of the environment-sensitive fluorophore acrylodan attached at either lobe of intact CaM and Ca^2+^ sensitive lobe-specific mutants, to Ca^2+^ binding to CaM free in solution or bound to RyR1, at steady and transient states. Our results suggest that Ca^2+^ binding causes a larger structural change on the low Ca^2^-affinity N-lobe *versus* high Ca^2+^-affinity C-lobe of free CaM at steady state. Transient kinetics measurements indicate that Ca^2+^ dissociation from the N-lobe is faster than from the C-lobe, while Ca^2+^ association to the C-lobe induces a faster structural change *versus* the N-lobe of CaM, as illustrated in Figure 7*A*. The primary kinetics of Ca^2+^ binding to CaM is maintained in the CaM/RyR1 complex, but the rate of Ca^2+^ dissociation from the N-lobe is ∼4 times slower *versus* the C-lobe. Ca^2+^ association to the C-lobe of CaM/RyR1 is modestly changed, but it is slower at the N-lobe. These changes in Ca^2+^ binding result in an increased Ca^2+^ affinity at the C-lobe of CaM/RyR1, and a marginal effect on Ca^2+^ affinity at the N-lobe of CaM/RyR1. In addition, the two Ca^2+^ binding sites of each lobe of CaM become more distinguishable after binding RyR1, suggesting that they assume more stable and distinct conformations in CaM/RyR1 complex versus free CaM. The higher Ca^2+^ association rate constants and larger fluorescence change in CaM *versus* CaM_C_ or CaM_N_ upon Ca^2+^ binding also indicate that cooperation between the two lobes is important for Ca^2+^ binding at both lobes. We propose that a rapid structural change of the C-lobe of CaM caused by Ca^2+^ association initiates CaM inhibition of RyR1 at high [Ca^2+^], and inhibition is completed by the slow structural change of the N-lobe. The relatively fast and large structural change of the N-lobe caused by Ca^2+^ dissociation enables the shift in the CaM binding position on RyR1 and its conversion to an RyR1 activator at low [Ca^2+^]. Both the C- and N-lobe of CaM are essential for this conversion. The model suggested by integration of these results with the relevant cryo-EM and FRET results is illustrated in Figure 7*B*. Based on the steady-state and transient kinetics of Ca^2+^ binding to CaM, we suggest that dysregulation of RyR1 by the oximimetic M124Q-CaM is caused by its intrinsically lower affinity for Ca^2+^, slower Ca^2+^ association rate, and lower affinity for RyR1. This work illustrates how kinetics of Ca^2+^ binding to CaM can be a valuable measure for mechanistic insight into disease-associated CaM mutants, or CaM regulation of its numerous targets.

## Experimental procedures

### Protein mutagenesis, expression, purification, and fluorescent labeling

CaM mutants with Cys substitutions for fluorescent labeling (T34C and T110C) and Ca^2+^ binding sites CaM_C_ (E31A/E67A) and CaM_N_ (E104A/E140A) were prepared by site-directed mutagenesis using Quikchange kit. For consistence with the literature, we did not include the initiator Met in the residue number assignment. The proteins were expressed and purified as described previously (51). The purified CaM and mutants were labeled with fluorescent probe acrylodan (^Acr^CaM). Fifteen-fold fluorescent dye dissolved in dimethyl sulfoxide (DMSO) was added to the protein (120 nM) in the buffer containing 100 mM Tris–HCl (pH 7.5) and 1.2 mM tris(2-carboxyethyl)phosphine hydrochloride (TCEP) and incubated with rotation at 25 °C for 2 h. Unreacted dye was removed using Zeba desalting columns according to manufacturer’s protocol (Thermo Fisher Scientific). Concentration of Acrylodan-labeled CaM was quantitated by absorbance max using the extinction coefficient of the dye, 12,900 M^−1^ cm^−1^. Protein concentration was determined by bicinchoninic acid (BCA) assay or gel densitometry.

### Isolation of SR vesicles

Crude SR membrane vesicles were isolated from homogenized porcine longissimus dorsi muscle by differential centrifugation. Heavy SR (HSR–enriched for RyR1), was isolated by fractionation of the crude SR using a discontinuous sucrose gradient (52). Samples were flash-frozen and stored at −80 °C. All experimental protocols were reviewed and approved by the University of Minnesota’s Institutional Animal Care and Use Committee, an Association for Assessment and Accreditation of Laboratory Animal Care institution.

### RyR1 purification

RyR1 was purified using procedures modified from previous reports (53, 54). HSR (140 mg) was incubated in solubilization buffer, which contained 20 mM Tris–HCl, 1 M NaCl, protease inhibitors (1 μg/ml aprotinin/leupeptin, 1 mM pepstatin A, 0.8 mM benzamidine, and 1 mM PMSF), 1 mM DTT, 1.5% CHAPS (VWR Life Science), 0.75% phosphatidylcholine (L-α-Lecithin from soybean, Sigma-Aldrich 429415), pH 7.5. The CHAPS-to-protein weight ratio was 12. Samples were incubated with solubilization buffer for 1 h at 4 °C with gentle stirring, and then centrifuged at 100,000g for 30 min at 4 °C. Supernatant was diluted in 50 mM Tris–HCl, protease inhibitors, 2 mM DTT, pH 7.5, and loaded onto a 5 ml glutathione agarose column (Thermo Fisher Scientific) preequilibrated with 20 ml cell lysate of GST-FKBP12.6 (derived from 1 L of BL21(DE3) pLysS cells overexpressing GST-FKBP12.6) and incubated overnight at 4 °C with gentle stirring. Briefly, the column was preequilibrated as follows: 10 ml of 50% glutathione agarose (Thermo Fisher Scientific) was washed with 10 column volumes of equilibration buffer (50 mM Tris, 150 mM NaCl, pH 7.5), incubated with cell lysate for 1 h at 4 °C with rocking, and washed with 10 column volumes of equilibration buffer. Following overnight incubation with solubilized HSR, the column was washed with 10 column volumes of wash buffer (50 mM Tris–HCl, 0.5% CHAPS, and 0.25% PC). RyR1 was eluted with 1.5 column volumes of wash buffer supplemented with 10 mM reduced glutathione. The eluate was spun down for 2 h at 250,000*g* at 4 °C. The pellet was resuspended in 20 mM Pipes, 1X protease inhibitors, 10% sucrose, pH 7.0, flash-frozen, and stored at −80 °C. Purified RyR1 concentration was determined by gel densitometry using HSR as a standard. The purified RyR1 was evaluated by the ^3^H-ryanodine binding assay described in our previous work (19).

### [^3^H]Ryanodine binding assay

Skeletal SR vesicles (1 mg/ml) were mixed with 300 nM CaM in a solution of 150 mM KCl, 5 mM GSH, 1 μg/ml aprotinin/leupeptin, 1 mM DTT, 1 mM EGTA, 65 μM or 1.02 mM CaCl_2_ (30 nM or 30 μM free Ca^2+^, respectively, as calculated using MaxChelator), 0.1 mg/ml bovine serum albumin, and 20 mM K-Pipes (pH 7.0). Binding of [^3^H]ryanodine (10 nM; PerkinElmer # NET950025UC) was determined upon 3-h incubation at 37 °C, followed by filtration through grade GF/B glass microfiber filters (Brandel Inc) using a 96-channel Brandel Harvester. In 4 ml of EcoLite scintillation mixture (MP Biomedicals), the [^3^H] retained on the filter was measured in a Perkin Elmer Tri-Carb 4810 scintillation counter. Data were reported as relative to the no CaM condition.

### Transient kinetics

Transient biochemical experiments with steady-state fluorescence (total fluorescence intensity) detection were performed on an Applied Photophysics SX.18MV stopped-flow spectrophotometer (Surry) under single-turnover conditions. All experiments were performed at 4 °C unless otherwise stated. The single-mix dead time for this instrument is 1.3 ms, calibrated using fluorescence enhancement of eight-hydroxyquinoline following Mg^2+^ binding under pseudo first-order kinetics conditions (55). In all experiments for free CaM, [^Acr^CaM] after mixing was 250 nM and the buffer 20 mM Mops and 30 mM NaCl at pH 7. All Ca^2+^ buffers (pH 7.0) were made using 1 M CaCl_2_ and 0.5 M EGTA (determined by MaxChelator) (bioworld and Thermo Fisher Scientific). In single-turnover experiments with RyR1, [RyR1] after mixing was 150 nM, so the ratio of CaM-binding sites: CaM was 2.4 in the buffer of 20 mM K-piperazine-N-N-bis(2-ethanesulfonic acid (K-Pipes), 150 mM KCl, 5 mM reduced glutathione, 1 g/ml aprotinin/leupeptin, 2 mM DTT, pH 7.0. ^Acr^CaM was excited at 375 nm and detected with a 400 nm long-pass filter. Nonlinear regression fitting of the kinetic traces of the Ca^2+^ binding to CaM was performed using the Applied Photophysics Pro-Data Software Suite version 4.2.12 (https://www.photophysics.com/) (56).

### Steady-state fluorescence measurement

Steady state fluorescence emission was recorded as a function of [Ca^2+^]. ^Acr^CaM (1 μM) was incubated with varying [Ca^2+^] in 20 mM Mops, 30 mM NaCl, pH 7, and the spectra were acquired from 400 nm to 600 nm (excitation, 375 nm) on Varian Cary Eclipse Fluorescence Spectrophotometer. Fluorescence peak-intensity and peak-wavelength were plotted *versus* [Ca^2+^]. The normalized fluorescence is calculated as [(F - F_min_)/(F_max_ - F_min_)].

### FRET competition assay

The inherent CaM of HSR was stripped off by incubating the HSR with CaMBP (300 nM) at 37 °C for 30 min. The binding of Alexa Fluor 488 (AF488) labeled FKBP12.6 (AF488-FKBP12.6) to HSR membranes (0.4 mg/ml) was accomplished by the incubation at 37 °C for 90 min in the buffer containing 150 mM KCl, 20 mM K-piperazine-N-N-bis(2-ethanesulfonic acid (K-Pipes; pH 7.0), 5 mM reduced glutathione, 0.1 mg/ml bovine serum albumin, 1 g/ml aprotinin/leupeptin, 1 mM DTT. AF488-85C-FKBP12.6 bound HSR were separated by centrifugation at 100,000*g* for 25 min, and the pellet was resuspended in the buffer and spun again. The pellets were then redissolved in the buffer to achieve a final HSR concentration of 1 mg/ml. These samples were then incubated with 100 nM Alexa Fluor 568 (AF568) labeled CaM (AF568–34C-CaM) and the indicated concentration range of unlabeled CaM mutants in the binding media containing 30 nM free Ca^2+^ or 30 μM free Ca^2+^. FRET efficiency of the samples was measured as the decrease of fluorescence intensity of AF488-85C-FKBP12.6 due to the competitive binding of unlabeled CaM to HSR (57).

### Circular dichroism measurements

CD spectra were recorded from 250 to 200 nm with a JASCO J-710 spectrophotometer coupled with a data processor. Spectra were recorded at 25 °C with a CaM concentration of 8 to 10 μM in a solution of 20 mM Mops and 30 mM NaCl, pH 7 using quartz cuvettes with a path length of 1.0 mm. Spectra were recorded with a scan speed of 20 nm/min, signal-averaged three times, and an equally signal-averaged solvent base line was subtracted.

## Data availability

All data are contained within the manuscript or supporting information.

## Supporting information

Fluorescence spectra of CaM labeled at T34 and T110 at different [Ca^2+^]; CD spectra of acrylodan labeled and unlabeled WT CaM, T34C, T110C, M124Q, and their lob-specific mutants; Effect of the labeling of CaM and its lobe-specific Ca^2+^-sensitive mutants on their regulation of RyR1; Representative two-exponential fit and residual of the kinetics data; Control measurements for Ca^2+^ association/dissociation with ^Acr110^CaM_1234;_ The fit of the initial fast change in the fluorescence of ^Acr34^CaM; Comparison of the amplitudes of fluorescence signal upon Ca^2+^ dissociation/association between intact CaM, CaM_N,_ and CaM_c_; Steady-State fluorescence emission spectra of ^Acr34^M124Q_C_ and ^Acr34^M124Q_N_; Electrophoretic mobility of WT, T34C, M124Q, and T34C/M124Q and its unlabeled and labeled lobe-specific Ca^2+^-sensitive mutants; Affinity of WT CaM and mutants and their lobe specific mutants to RyR1. Summary of the apparent K_D_ values obtained by different methods; Comparison of the off-rates of the Ca^2+^ dissociation from the CaM, and its C- and N-lobes obtained by different methods (2, 4, 7, 8, 9, 11, 21, 23, 25, 26, 34, 58).

## Conflict of interest

Razvan Cornea is currently an employee of the National Institutes of Health. This work was conducted during his previous employment, at the University of Minnesota – Twin Cities. The opinions expressed in this article are the author’s own and do not reflect the view of the National Institutes of Health, the Department of Health and Human Services, or the United States government.

## References

[1] Seiler S., Wegener A.D., Whang D.D., Hathaway D.R., Jones L.R. (1984). High molecular weight proteins in cardiac and skeletal muscle junctional sarcoplasmic reticulum vesicles bind calmodulin, are phosphorylated, and are degraded by Ca^2+^-activated protease. J. Biol. Chem..

[2] Babu Y.S., Sack J.S., Greenhough T.J., Bugg C.E., Means A.R., Cook W.J. (1985). Three-dimensional structure of calmodulin. Nature.

[3] Linse S., Helmersson A., Forsén S. (1991). Calcium binding to calmodulin and its globular domains. J. Biol. Chem..

[4] VanScyoc W.S., Sorensen B.R., Rusinova E., Laws W.R., Ross J.B., Shea M.A. (2002). Calcium binding to calmodulin mutants monitored by domain-specific intrinsic phenylalanine and tyrosine fluorescence. Biophys. J..

[5] Rodney G.G., Williams B.Y., Strasburg G.M., Beckingham K., Hamilton S.L. (2000). Regulation of RYR1 activity by Ca^2+^ and calmodulin. Biochemistry.

[6] Tripathy A., Xu L., Mann G., Meissner G. (1995). Calmodulin activation and inhibition of skeletal muscle Ca^2+^ release channel (ryanodine receptor). Biophys. J..

[7] Buratti R., Prestipino G., Menegazzi P., Treves S., Zorzato F. (1995). Calcium dependent activation of skeletal muscle Ca^2+^ release channel (ryanodine receptor) by calmodulin. Biochem. Biophys. Res. Commun..

[8] Fuentes O., Valdivia C., Vaughan D., Coronado R., Valdivia H.H. (1994). Calcium-dependent block of ryanodine receptor channel of swine skeletal muscle by direct binding of calmodulin. Cell Calcium.

[9] Gangopadhyay J.P., Grabarek Z., Ikemoto N. (2004). Fluorescence probe study of Ca^2+^-dependent interactions of calmodulin with calmodulin-binding peptides of the ryanodine receptor. Biochem. Biophys. Res. Commun..

[10] Newman R.A., Sorensen B.R., Kilpatrick A.M., Shea M.A. (2014). Calcium-dependent energetics of calmodulin domain interactions with regulatory regions of the Ryanodine Receptor Type 1 (RyR1). Biophys. Chem..

[11] Fruen B.R., Balog E.M., Schafer J., Nitu F.R., Thomas D.D., Cornea R.L. (2005). Direct detection of calmodulin tuning by ryanodine receptor channel targets using a Ca^2+^-sensitive acrylodan-labeled calmodulin. Biochemistry.

[12] Xiong L.W., Newman R.A., Rodney G.G., Thomas O., Zhang J.Z., Persechini A. (2002). Lobe-dependent regulation of ryanodine receptor type 1 by calmodulin. J. Biol. Chem..

[13] Boschek C.B., Jones T.E., Squier T.C., Bigelow D.J. (2007). Calcium occupancy of N-terminal sites within calmodulin induces inhibition of the ryanodine receptor calcium release channel. Biochemistry.

[14] Gong D., Chi X., Wei J., Zhou G., Huang G., Zhang L. (2019). Modulation of cardiac ryanodine receptor 2 by calmodulin. Nature.

[15] Woll K.A., Haji-Ghassemi O., Van Petegem F. (2021). Pathological conformations of disease mutant Ryanodine Receptors revealed by cryo-EM. Nat. Commun..

[16] Huang X., Fruen B., Farrington D.T., Wagenknecht T., Liu Z. (2012). Calmodulin-binding locations on the skeletal and cardiac ryanodine receptors. J. Biol. Chem..

[17] Melville Z., Dridi H., Yuan Q., Reiken S., Wronska A., Liu Y. (2022). A drug and ATP binding site in type 1 ryanodine receptor. Structure.

[18] Maximciuc A.A., Putkey J.A., Shamoo Y., MacKenzie K.R. (2006). Complex of calmodulin with a ryanodine receptor target reveals a novel, flexible binding mode. Structure.

[19] McCarthy M.R., Savich Y., Cornea R.L., Thomas D.D. (2020). Resolved structural states of calmodulin in regulation of skeletal muscle calcium release. Biophys. J..

[20] Rebbeck R.T., Svensson B., Zhang J., Samso M., Thomas D.D., Bers D.M. (2024). Kinetics and mapping of Ca-driven calmodulin conformations on skeletal and cardiac muscle ryanodine receptors. Nat. Commun..

[21] Andersson T., Drakenberg T., Forsén S., Thulin E. (1982). Characterization of the Ca^2+^ binding sites of calmodulin from bovine testis using ^43^Ca and ^113^Cd NMR. Eur. J. Biochem..

[22] Ikura M., Hiraoki T., Hikichi K., Mikuni T., Yazawa M., Yagi K. (1983). Nuclear magnetic resonance studies on calmodulin: calcium-induced conformational change. Biochemistry.

[23] Malencik D.A., Anderson S.R., Shalitin Y., Schimerlik M.I. (1981). Rapid kinetic studies on calcium interactions with native and fluorescently labeled calmodulin. Biochem. Biophys. Res. Commun..

[24] Schimerlik M.I., Malencik D.A., Anderson S.R., Shalitin Y. (1982). Rapid kinetic studies of calmodulin interactions with calcium and troponin I as monitored by anthroylcholine fluorescence. Biochem. Biophys. Res. Commun..

[25] Bayley P., Ahlström P., Martin S.R., Forsén S. (1984). The kinetics of calcium binding to calmodulin: Quin 2 and ANS stopped-flow fluorescence studies. Biochem. Biophys. Res. Commun..

[26] Faas G.C., Raghavachari S., Lisman J.E., Mody I. (2011). Calmodulin as a direct detector of Ca^2+^ signals. Nat. Neurosci..

[27] Park H.Y., Kim S.A., Korlach J., Rhoades E., Kwok L.W., Zipfel W.R. (2008). Conformational changes of calmodulin upon Ca^2+^ binding studied with a microfluidic mixer. Proc. Natl. Acad. Sci. U. S. A..

[28] Chin D., Means A.R. (2000). Calmodulin: a prototypical calcium sensor. Trends Cell Biol..

[29] Wu X., Bers D.M. (2007). Free and bound intracellular calmodulin measurements in cardiac myocytes. Cell Calcium.

[30] Prendergast F.G., Meyer M., Carlson G.L., Iida S., Potter J.D. (1983). Synthesis, spectral properties, and use of 6-acryloyl-2-dimethylaminonaphthalene (Acrylodan). A thiol-selective, polarity-sensitive fluorescent probe. J. Biol. Chem..

[31] Balog E.M., Norton L.E., Bloomquist R.A., Cornea R.L., Black D.J., Louis C.F. (2003). Calmodulin oxidation and methionine to glutamine substitutions reveal methionine residues critical for functional interaction with ryanodine receptor-1. J. Biol. Chem..

[32] O’Neil K.T., DeGrado W.F. (1990). How calmodulin binds its targets: sequence independent recognition of amphiphilic alpha-helices. Trends Biochem. Sci..

[33] Balog E.M., Norton L.E., Thomas D.D., Fruen B.R. (2006). Role of calmodulin methionine residues in mediating productive association with cardiac ryanodine receptors. Am. J. Physiol. Heart Circ. Physiol..

[34] Kilhoffer M.C., Kubina M., Travers F., Haiech J. (1992). Use of engineered proteins with internal tryptophan reporter groups and perturbation techniques to probe the mechanism of ligand-protein interactions: investigation of the mechanism of calcium binding to calmodulin. Biochemistry.

[35] Pedigo S., Shea M.A. (1995). Discontinuous equilibrium titrations of cooperative calcium binding to calmodulin monitored by 1-D ^1^H-nuclear magnetic resonance spectroscopy. Biochemistry.

[36] Shea M.A., Verhoeven A.S., Pedigo S. (1996). Calcium-induced interactions of calmodulin domains revealed by quantitative thrombin footprinting of Arg37 and Arg106. Biochemistry.

[37] Sorensen B.R., Shea M.A. (1998). Interactions between domains of apo calmodulin alter calcium binding and stability. Biochemistry.

[38] Wriggers W., Mehler E., Pitici F., Weinstein H., Schulten K. (1998). Structure and dynamics of calmodulin in solution. Biophys. J..

[39] Chen B., Mayer M.U., Markillie L.M., Stenoien D.L., Squier T.C. (2005). Dynamic motion of helix A in the amino-terminal domain of calmodulin is stabilized upon calcium activation. Biochemistry.

[40] Boschek C.B., Squier T.C., Bigelow D.J. (2007). Disruption of interdomain interactions via partial calcium occupancy of calmodulin. Biochemistry.

[41] Fruen B.R., Black D.J., Bloomquist R.A., Bardy J.M., Johnson J.D., Louis C.F. (2003). Regulation of the RYR1 and RYR2 Ca2+ release channel isoforms by Ca^2+^-insensitive mutants of calmodulin. Biochemistry.

[42] VanScyoc W.S., Newman R.A., Sorensen B.R., Shea M.A. (2006). Calcium binding to calmodulin mutants having domain-specific effects on the regulation of ion channels. Biochemistry.

[43] Rivard B.S., Rogers M.S., Marell D.J., Neibergall M.B., Chakrabarty S., Cramer C.J. (2015). Rate-determining attack on substrate precedes rieske cluster oxidation during cis-dihydroxylation by benzoate dioxygenase. Biochemistry.

[44] Jaren O.R., Kranz J.K., Sorensen B.R., Wand A.J., Shea M.A. (2002). Calcium-induced conformational switching of paramecium calmodulin provides evidence for domain coupling. Biochemistry.

[45] Maune J.F., Klee C.B., Beckingham K. (1992). Ca^2+^ binding and conformational change in two series of point mutations to the individual Ca^2+^-binding sites of calmodulin. J. Biol. Chem..

[46] Samso M., Wagenknecht T. (2002). Apocalmodulin and Ca^2+^-calmodulin bind to neighboring locations on the ryanodine receptor. J. Biol. Chem..

[47] Rodney G.G., Krol J., Williams B., Beckingham K., Hamilton S.L. (2001). The carboxy-terminal calcium binding sites of calmodulin control calmodulin's switch from an activator to an inhibitor of RYR1. Biochemistry.

[48] Tadross M.R., Dick I.E., Yue D.T. (2008). Mechanism of local and global Ca^2+^ sensing by calmodulin in complex with a Ca^2+^ channel. Cell.

[49] McCarthy M.R., Thompson A.R., Nitu F., Moen R.J., Olenek M.J., Klein J.C. (2015). Impact of methionine oxidation on calmodulin structural dynamics. Biochem. Biophys. Res. Commun..

[50] Nelson S.E.D., Weber D.K., Rebbeck R.T., Cornea R.L., Veglia G., Thomas D.D. (2020). Met125 is essential for maintaining the structural integrity of calmodulin's C-terminal domain. Sci. Rep..

[51] Balog E.M., Lockamy E.L., Thomas D.D., Ferrington D.A. (2009). Site-specific methionine oxidation initiates calmodulin degradation by the 20S proteasome. Biochemistry.

[52] Cornea R.L., Nitu F.R., Samso M., Thomas D.D., Fruen B.R. (2010). Mapping the ryanodine receptor FK506-binding protein subunit using fluorescence resonance energy transfer. J. Biol. Chem..

[53] Wei R., Wang X., Zhang Y., Mukherjee S., Zhang L., Chen Q. (2016). Structural insights into Ca^2+^-activated long-range allosteric channel gating of RyR1. Cell Res..

[54] Zalk R., Clarke O.B., des Georges A., Grassucci R.A., Reiken S., Mancia F. (2015). Structure of a mammalian ryanodine receptor. Nature.

[55] Brissette P., Ballou D.P., Massey V. (1989). Determination of the dead time of a stopped-flow fluorometer. Anal. Biochem..

[56] Brazeau B.J., Lipscomb J.D. (2000). Kinetics and activation thermodynamics of methane monooxygenase compound Q formation and reaction with substrates. Biochemistry.

[57] Rebbeck R.T., Nitu F.R., Rohde D., Most P., Bers D.M., Thomas D.D. (2016). S100A1 protein does not compete with calmodulin for ryanodine receptor binding but structurally alters the ryanodine receptor·calmodulin complex. J. Biol. Chem..

[58] Kincaid R.L., Vaughan M., Osborne J.C., Tkachuk V.A. (1982). Ca^2+^-dependent interaction of 5-dimethylaminonaphthalene-1-sulfonyl-calmodulin with cyclic nucleotide phosphodiesterase, calcineurin, and troponin I. J. Biol. Chem..

