# Supplementary material for: Kinetics insight into the roles of the N- and C-lobes of calmodulin in RyR1 channel regulation
Source: J Biol Chem. 2025 Feb 2;301(3):108258. doi: 10.1016/j.jbc.2025.108258 (PMC11923823; doi:10.1016/j.jbc.2025.108258)
Supplement: Supporting information [file mmc1.pdf]

Supporting information

Kinetics insights into the roles of the N- and C-lobes of calmodulin in RyR1 channel regulation

Jingyan Zhang[1], Levy M. Treinen[1], Skylar J. Mast[1], Megan R. McCarthy[1], Bengt Svensson[1], David D. Thomas[1], Razvan L. Cornea[1]*

[1]Department of Biochemistry, Molecular Biology, and Biophysics, University of Minnesota, Minneapolis, MN, USA

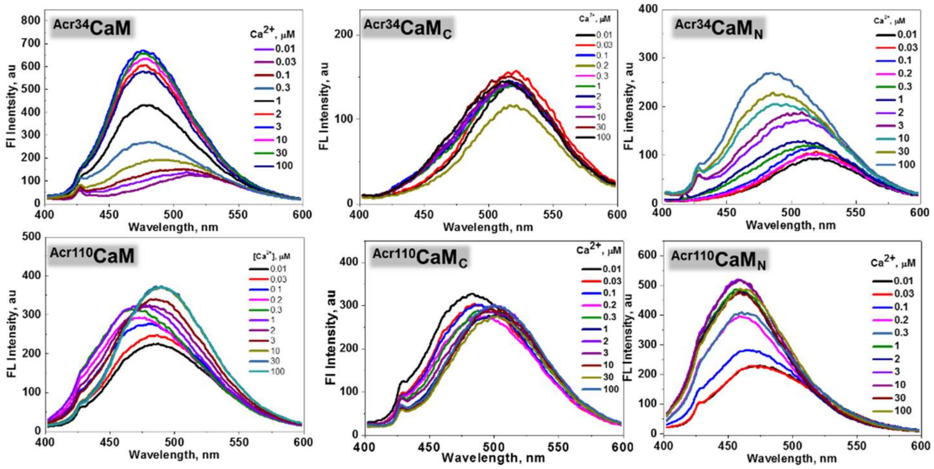

Figure S1. Full fluorescence spectra of the labeled CaMs at designed [Ca^{2+}]. The spectra were acquired in a solution of 20 mM MOPS, 30 mM NaCl, pH=7 at 21°C.

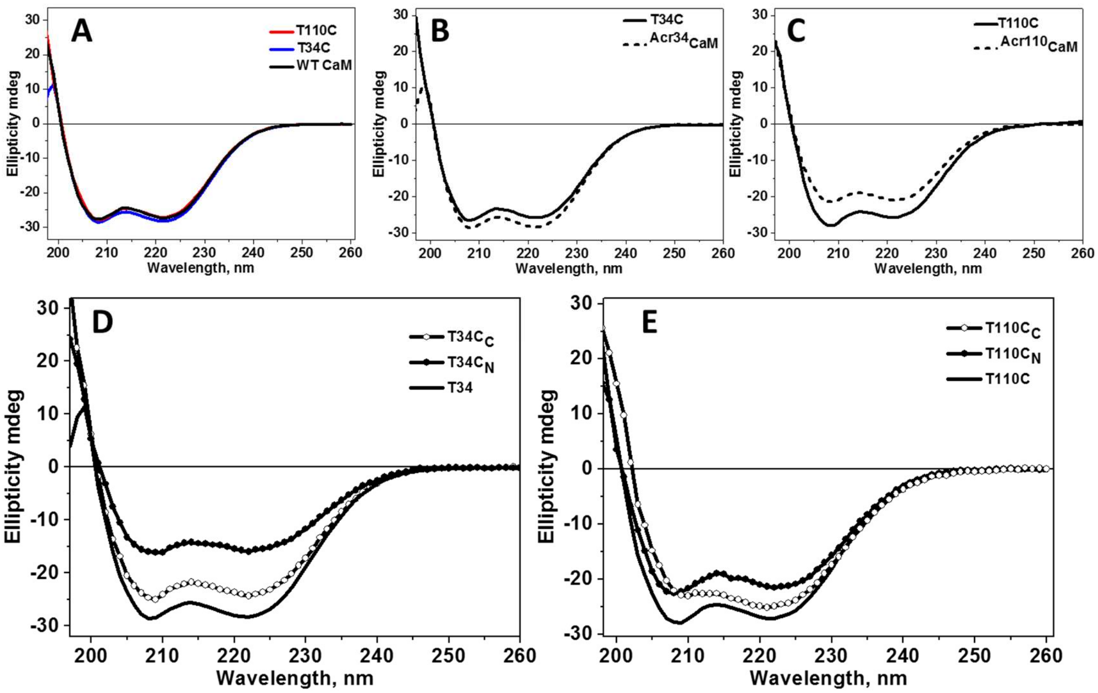

Figure S2. CD spectra of WT-, T34C-, and T110C-CaM (A), overlay of the acrylodan-labeled (dotted line) and unlabeled (solid line) T34C-CaM (B) and T110C-CaM (C), and comparison of the lobe-specific Ca^{2+}-sensitive mutants of T34C-CaM (D) and T110C-CaM (E). Spectra were acquired with 10 μM protein in a solution of 20 mM MOPS, 30 mM NaCl, pH=7 at 21°C.

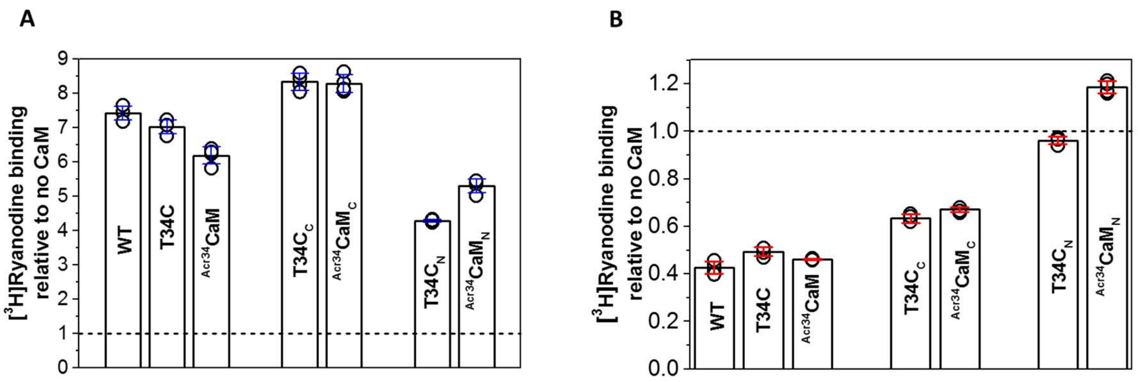

Figure S3. [^{3}H]Ryanodine binding activity of RyR1 with WT-CaM, T34C, T34C$_C$, T34C$_N$, and their acrylodan-labeled forms at (A) 100 nM Ca^{2+}, (B) 30 µM Ca^{2+}.

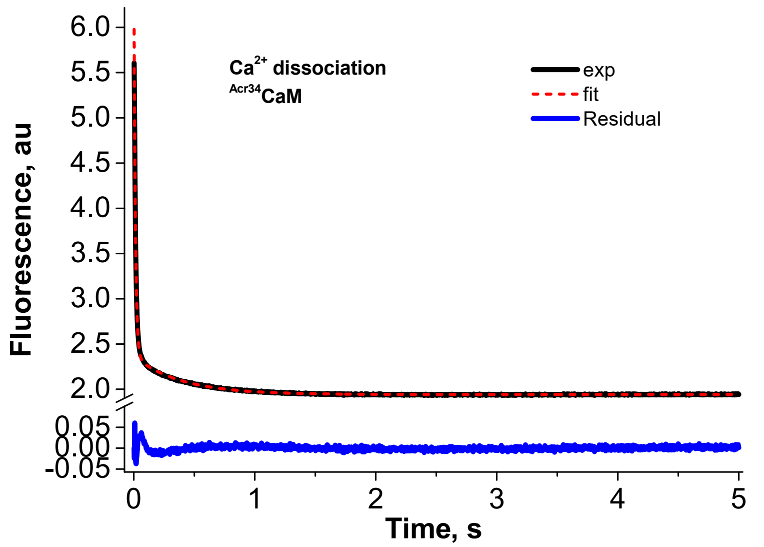

Figure S4. Representative time-course (black) and two-exponential fit (red dotted line) and residual (blue trace) of Ca^{2+} dissociation from ^{Acr34}CaM. ^{Acr34}CaM (0.5 µM) pre-saturated with 30 µM Ca^{2+} was rapidly mixed with an equal volume of 2 mM EGTA in a stopped-flow apparatus at 4ºC in 20 mM MOPS, 30 mM NaCl, pH 7. Data were fitted using the Applied Photophysics Pro-Data Software Suite (version 4.2.12).

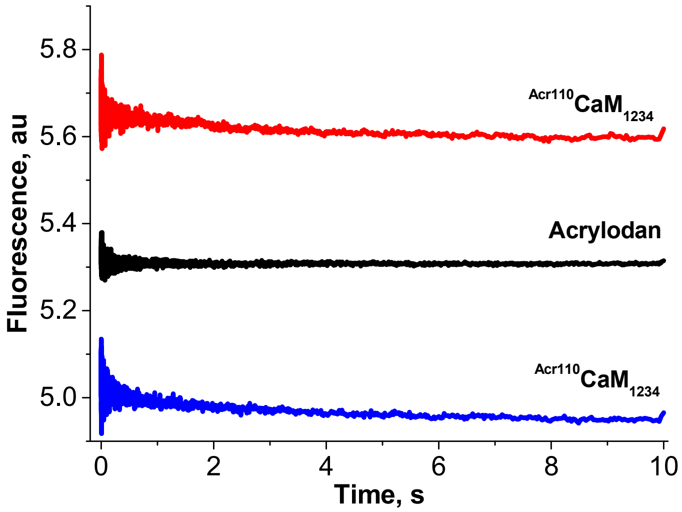

Figure S5. Control measurements for Ca^{2+} association and dissociation from $^{Acr110}CaM_{1234}$, in which the Ca^{2+} binding sites of the two lobes of CaM were ablated. Red trace: $^{Acr110}CaM_{1234}$ (0.5 µM) was rapidly mixed with an equal volume of 20 µM Ca^{2+} in a stopped-flow apparatus at 4°C in 20 mM MOPS, 30 mM NaCl, pH 7. Blue trace: $^{Acr110}CaM_{1234}$ was mixed with 2 mM EGTA as Ca^{2+} dissociation control. Black trace: Free acrylodan was mixed with 2 mM EGTA.

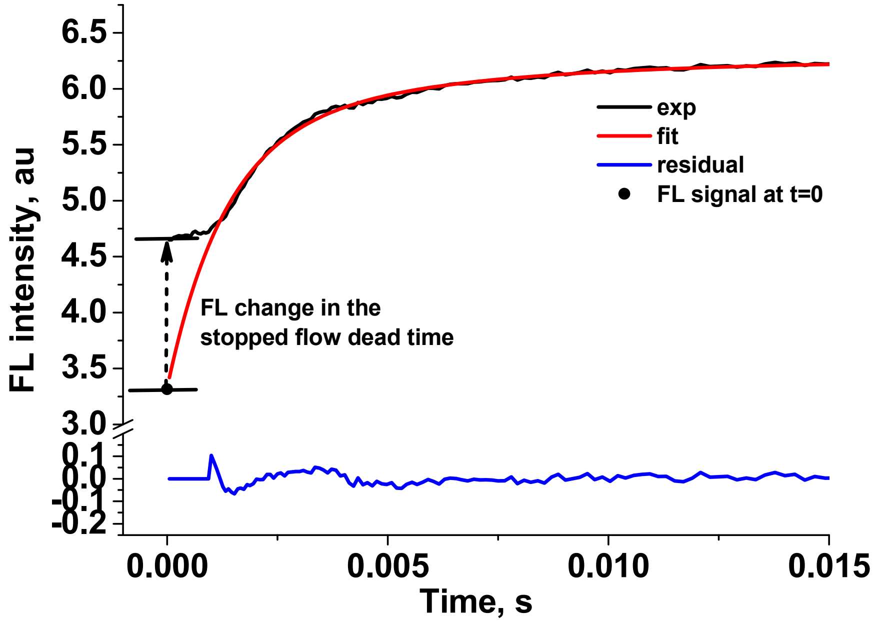

Figure S6. Fitting the initial fast change of the fluorescence intensity of ^{Acr34}CaM (0.5 µM), mixed with 20 µM Ca^{2+} in a stopped-flow instrument, using a buffer containing 20 mM MOPS and 30 mM NaCl, pH 7, 4°C. The fit was started from 0.001s. The change in fluorescence intensity occurring in the instrument dead-time is indicated by the arrow.

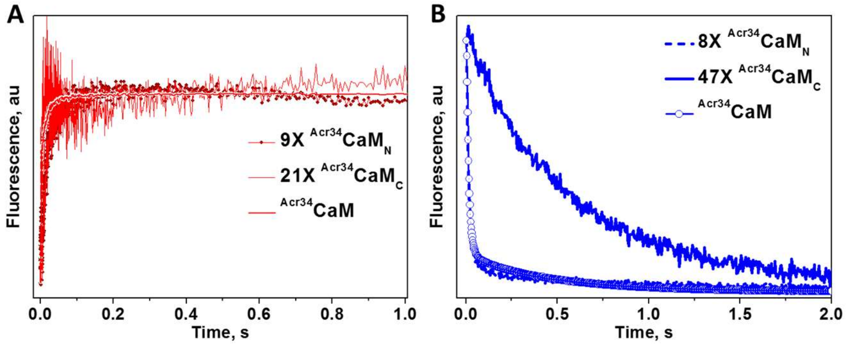

Figure S7. Comparison of the amplitudes of fluorescence changes upon Ca^{2+} association (A) and dissociation (B) between intact CaM, CaM_N, and CaM_C (data correspond to the red and blue traces of the left column in Figure 3). To facilitate comparison with intact CaM, traces corresponding to CaM_N, and CaM_C were multiplied by the factor indicated in the figure.

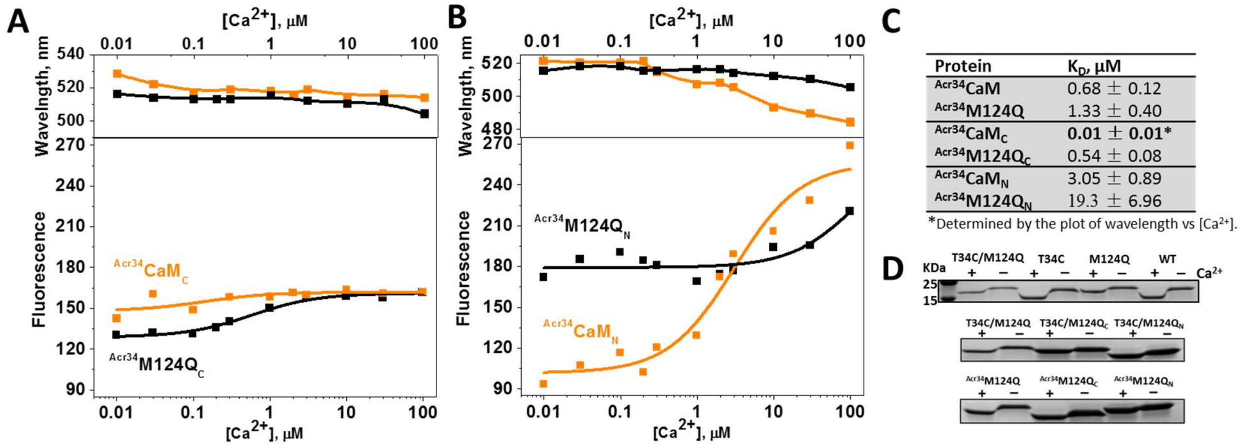

Protein	K_D, μM
^{Acr34}CaM	0.68 ± 0.12
^{Acr34}M124Q	1.33 ± 0.40
^{Acr34}CaM$_C$	**0.01 ± 0.01***
^{Acr34}M124Q$_C$	0.54 ± 0.08
^{Acr34}CaM$_N$	3.05 ± 0.89
^{Acr34}M124Q$_N$	19.3 ± 6.96

*Determined by the plot of wavelength vs [Ca^{2+}].

Figure S8. Representative steady-state fluorescence measurements of ^{Acr34}M124Q$_C$ (A) and ^{Acr34}M124Q$_N$ (B) (black plots) and corresponding controls ^{Acr34}CaM$_C$ and ^{Acr34}CaM$_N$ (orange plots) in a solution of 20 mM MOPS, 30 mM NaCl (pH 7), containing 0.01 to 100 μM [Ca^{2+}] as indicated. The top panels display the peak-wavelength of the fluorescence spectrum vs. [Ca^{2+}]. The bottom panels display the peak-intensity vs. [Ca^{2+}]. Solid lines in the bottom two panels represent fits using the Hill function. (C) Apparent K_D values obtained from fitting the curves illustrated in A and B, and Figure 5C; means ± SD, n = 3. (D) SDS-PAGE of WT, T34C, M124Q, T34C/M124Q (top strip) and their lobe-specific Ca^{2+} sensitive mutants unlabeled (middle strip) and labeled T34C/M124Q, T34C/M124Q$_C$, and T34C/M124Q$_N$ (bottom strip). Gels were run as described in "Experimental Procedures," after incubation in sample buffer containing either 5 mM Ca^{2+} (+) or 5 mM EGTA (-).

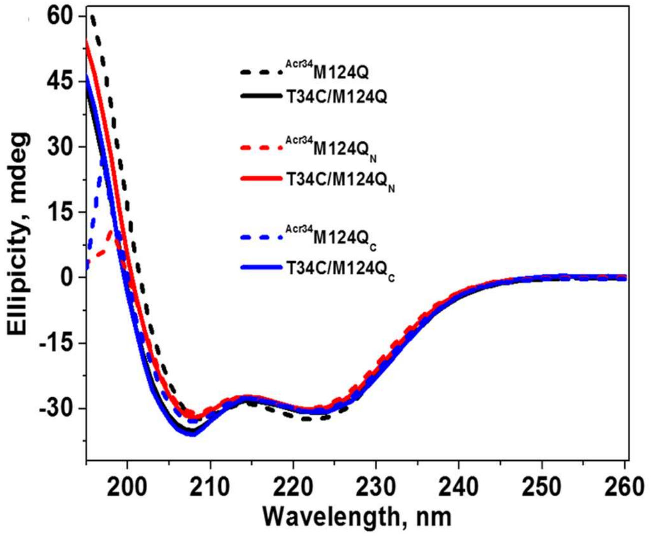

Figure S9. CD spectra of CaM mutants T34C/M124Q, T34C/M124Q$_C$, and T34C/M124Q$_N$ and the corresponding acrylodan-labeled proteins. Spectra were acquired using 10 μM protein in a buffer of 20 mM MOPS, 30 mM NaCl, pH=7 at 21°C.

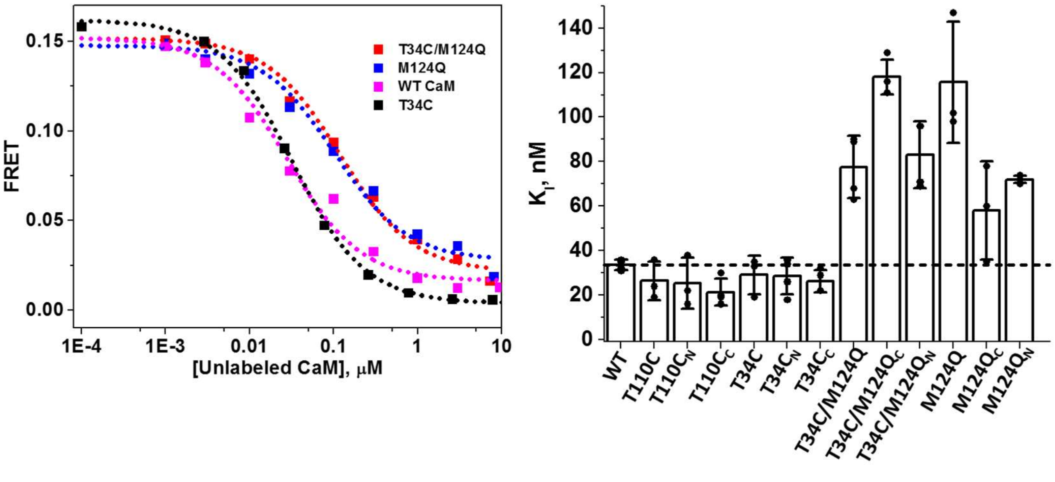

Figure S10. (A) Representative FRET competition binding of unlabeled CaM variants (as indicated in the legend) to HSR-pretreated with AF488-FKBP12.6 (donor) and AF568-34C-CaM (acceptor). For detailed experimental details, refer to "Experimental Procedures". (B) Summary of K_I values for curves similar to those in panel A, for the indicated CaM variants. K_I values were determined by fitting the FRET competition data to the Hill function and are represented as means ± SD (n = 3).

Table S1. Summary of K_D values obtained by fitting plots of fluorescence intensity and wavelength vs [Ca^{2+}] in steady state fluorescence measurements with fluorescent dye-labeled CaM, tryptophan-engineered CaM, or WT-CaM (using the intrinsic tyrosine and phenylalanine).

Protein	K_D, μM	Reference
^{Acr34}CaM	0.7 ± 0.12	This work
^{Acr34}CaM$_C$	0.01±0.01[6]	
^{Acr34}CaM$_N$	3.1 ± 0.9	
^{Acr110}CaM	0.1 ± 0.03/<br>3.8 ± 0.7	This work
^{Acr110}CaM$_C$	0.25±0.06[6]	
^{Acr110}CaM$_N$	0.2 ± 0.02	
CaM[1]	0.7	(1)
CaM[2]	2.1 ± 0.2	(2)
CaM$_N$	5.2 ± 0.6	
CaMc	1.2 ± 0.1	
CaM[3]	2.5± 0.3	(3)
CaM[4]	2.2	(4)
CaM[5]	2.2 - 3.85	(5)
^{Acr26}CaM	2.7 ± 0.8	(6)

1. CaM labeled at T34C with Badan.
2. CaM Labeled at T34C or T110C with N-(1-Pyrene) maleimide.
3. K_D determined from measurements of intrinsic tyrosine fluorescence of CaM.
4. CaM labeled with dansyl-chloride.
5. K_D determined from measurements of fluorescence of a tryptophan-engineered CaM mutant.
6. K_D was obtained by fitting the wavelength shift of the fluorescence peak vs. [Ca^{2+}].

Table S2. Comparison of the off-rates (k_{off}) for Ca^{2+} dissociation from each lobe of CaM, using different detection methods.

k_{off} s^{-1}	^{Acr110}CaM 4°C	^{Acr110}CaM 25°C	^{Acr34}CaM 4°C	^{43}Ca-NMR 23°C (7)	^{1}H-NMR 23°C (8)	CaM 11°C (9)	CaM 21°C (10)	CaM 21°C (10,11)	CaM 35°C (12)
C-lobe	2.2	25.7	2.4	20-50	<50	2.2	10	~10	8.7E3-9.6
N-lobe	55	396	67	~1000	>600	356	0.3		5.2E5-1.2E3

References:

1. Gangopadhyay, J. P., Grabarek, Z., and Ikemoto, N. (2004) Fluorescence probe study of Ca2+-dependent interactions of calmodulin with calmodulin-binding peptides of the ryanodine receptor. *Biochemical and biophysical research communications* **323**, 760-768
2. Boschek, C. B., Squier, T. C., and Bigelow, D. J. (2007) Disruption of Interdomain Interactions via Partial Calcium Occupancy of Calmodulin. *Biochemistry* **46**, 4580-4588
3. VanScyoc, W. S., Sorensen, B. R., Rusinova, E., Laws, W. R., Ross, J. B., and Shea, M. A. (2002) Calcium binding to calmodulin mutants monitored by domain-specific intrinsic phenylalanine and tyrosine fluorescence. *Biophys J* **83**, 2767-2780
4. Kincaid, R. L., Vaughan, M., Osborne, J. C., Jr., and Tkachuk, V. A. (1982) Ca2+-dependent interaction of 5-dimethylaminonaphthalene-1-sulfonyl-calmodulin with cyclic nucleotide

phosphodiesterase, calcineurin, and troponin I. *The Journal of biological chemistry* **257**, 10638-10643
5. Kilhoffer, M. C., Kubina, M., Travers, F., and Haiech, J. (1992) Use of engineered proteins with internal tryptophan reporter groups and perturbation techniques to probe the mechanism of ligand-protein interactions: investigation of the mechanism of calcium binding to calmodulin. *Biochemistry* **31**, 8098-8106
6. Fruen, B. R., Balog, E. M., Schafer, J., Nitu, F. R., Thomas, D. D., and Cornea, R. L. (2005) Direct detection of calmodulin tuning by ryanodine receptor channel targets using a Ca2+-sensitive acrylodan-labeled calmodulin. *Biochemistry* **44**, 278-284
7. Andersson, T., Drakenberg, T., ForsÉN, S., and Thulin, E. (1982) Characterization of the Ca2+ Binding Sites of Calmodulin from Bovine Testis Using 43Ca and 113Cd NMR. *European Journal of Biochemistry* **126**, 501-505
8. Ikura, M., Hiraoki, T., Hikichi, K., Mikuni, T., Yazawa, M., and Yagi, K. (1983) Nuclear magnetic resonance studies on calmodulin: calcium-induced conformational change. *Biochemistry* **22**, 2573-2579
9. Bayley, P., Ahlstrom, P., Martin, S. R., and Forsen, S. (1984) The kinetics of calcium binding to calmodulin: Quin 2 and ANS stopped-flow fluorescence studies. *Biochemical and biophysical research communications* **120**, 185-191
10. Malencik, D. A., Anderson, S. R., Shalitin, Y., and Schimerlik, M. I. (1981) Rapid kinetic studies on calcium interactions with native and fluorescently labeled calmodulin. *Biochemical and biophysical research communications* **101**, 390-395
11. Schimerlik, M. I., Malencik, D. A., Anderson, S. R., and Shalitin, Y. (1982) Rapid kinetic studies of calmodulin interactions with calcium and troponin I as monitored by anthroylcholine fluorescence. *Biochemical and biophysical research communications* **106**, 1331-1339
12. Faas, G. C., Raghavachari, S., Lisman, J. E., and Mody, I. (2011) Calmodulin as a direct detector of Ca2+ signals. *Nature neuroscience* **14**, 301-304
